# Prediction of Structure and Molecular Interaction with DNA of BvrR, a Virulence-Associated Regulatory Protein of *Brucella*

**DOI:** 10.3390/molecules24173137

**Published:** 2019-08-29

**Authors:** Edgar A. Ramírez-González, Martha C. Moreno-Lafont, Alfonso Méndez-Tenorio, Mario E. Cancino-Díaz, Iris Estrada-García, Rubén López-Santiago

**Affiliations:** 1 Departamento de Inmunología, Escuela Nacional de Ciencias Biológicas, Instituto Politécnico Nacional, Ciudad de México 11340, Mexico; 2 Laboratorio de Biotecnología y Bioinformática Genómica, Escuela Nacional de Ciencias Biológicas, Instituto Politécnico Nacional, Ciudad de México 11340, Mexico

**Keywords:** BvrR, protein structure prediction, Gibbs sampling, protein-DNA docking, MD simulation

## Abstract

Brucellosis, also known as “undulant fever” is a zoonotic disease caused by *Brucella*, which is a facultative intracellular bacterium. Despite efforts to eradicate this disease, infection in uncontrolled domestic animals persists in several countries and therefore transmission to humans is common. *Brucella* evasion of the innate immune system depends on its ability to evade the mechanisms of intracellular death in phagocytic cells. The BvrR-BvrS two-component system allows the bacterium to detect adverse conditions in the environment. The BvrS protein has been associated with genes of virulence factors, metabolism, and membrane transport. In this study, we predicted the DNA sequence recognized by BvrR with Gibbs Recursive Sampling and identified the three-dimensional structure of BvrR using I-TASSER suite, and the interaction mechanism between BvrR and DNA with Protein-DNA docking and molecular dynamics (MD) simulation. Based on the Gibbs recursive Sampling analysis, we found the motif AAHTGC (H represents A, C, and T nucleotides) as a possible sequence recognized by BvrR. The docking and EMD simulation results showed that C-terminal effector domain of BvrR protein is likely to interact with AAHTGC sequence. In conclusion, we predicted the structure, recognition motif, and interaction of BvrR with DNA.

## 1. Introduction

Brucellosis is one of the most common zoonoses in the world and is caused by microorganisms of the genus *Brucella*, which can be transmitted directly or indirectly to humans. The disease is transmitted from animals to humans through genital excretions and contaminated milk, the greatest source of human infection [1,2]. Infection can occur through the skin or mucous membrane lesions and by inhalation of contaminated dust or aerosols (an estimated dose of 10 to 100 microorganisms is enough to establish aerial infection) and is one of the most common laboratory-acquired infections [3,4]. Infections by *B. abortus* and *B. suis* usually affect occupational groups (veterinarians, farmers, and abattoir workers), whereas that caused by *B. melitensis* is more frequent in the community [5,6]. The disease presents polymorphous clinical manifestations and is often asymptomatic [7]. Acute brucellosis manifests as a febrile disease. The fever increases progressively until reaching a plateau that lasts for several days and then descends slowly; this first febrile cycle can precede other shorter successive febrile cycles [8]. Brucellosis without treatment progresses to a disabling chronic disease with severe complications, such as central nervous system (CNS) affectations, osteomyelitis, keratitis, and endocarditis. The susceptibility to brucellosis in humans depends on the immunological state of the person, the route of infection, the size of the inoculum, and the virulence of the species [2].

*Brucella* possesses mechanisms of immune response evasion that allow it to be an intracellular parasite in macrophages, monocytes, and epithelial cells [9]. For the internalization, the bacteria’s protein Hsp60 and lipopolysaccharide (LPS) are recognized by proteins located on the lipid rafts of the cell membrane [10]. Once internalized, the bacterium resides in Brucella-containing vacuoles (BCVs) [11] that interact with multivesicular bodies (MVB) and early compartments of the endocytic pathway [12,13]. Then, they interact with late endocytic organelles [11,13] and partially fuse with the lysosomes [14]. Finally, the BCVs intercept the endoplasmic reticulum exit sites, fuse with them, and form an organelle that is permissive to replication [11]. The interaction between BCVs and endosomes and lysosomes is controlled to allow acidification, which activates the BvrS/BvrR two-component system, composed of a transmembrane histidine kinase sensor (BvrS) and a cytosolic response regulator (BvrR). This system controls the expression of outer membrane proteins, as Omp22 and Omp25, and the structure of the LPS [15,16,17,18]; it also controls the expression of the *virB* operon coding for the type IV secretion system, which secretes bacterial factors that modulate the maturation of the BCV [19,20]. The BvrS/BvrR two-component system is essential to the detection of changes in the phagosomal environment and the modification of the extracellular lifestyle into an intracellular one [16,21]. The two-component system works through the transduction of environmental signals. While acidity is essential, other factors also contribute and are first detected by the histidine kinase sensor, which is autophosphorylated in a histidine residue. The phosphate group is transferred to an aspartate residue in the regulatory protein, which mediates the changes in gene expression [21]. This system also provides *Brucella* with resistance to polycationic detergents and increases permeability to surfactants, so it has been proposed that some molecular characteristics of the outer membrane are under the control of the BvrR/BvrS system [22,23]. Mutant strains in the BvrR/BvrS system are avirulent in mouse, show a lower invasive capacity in macrophages and HeLa cells, and are unable to replicate intracellularly [15].

**Figure 1:**
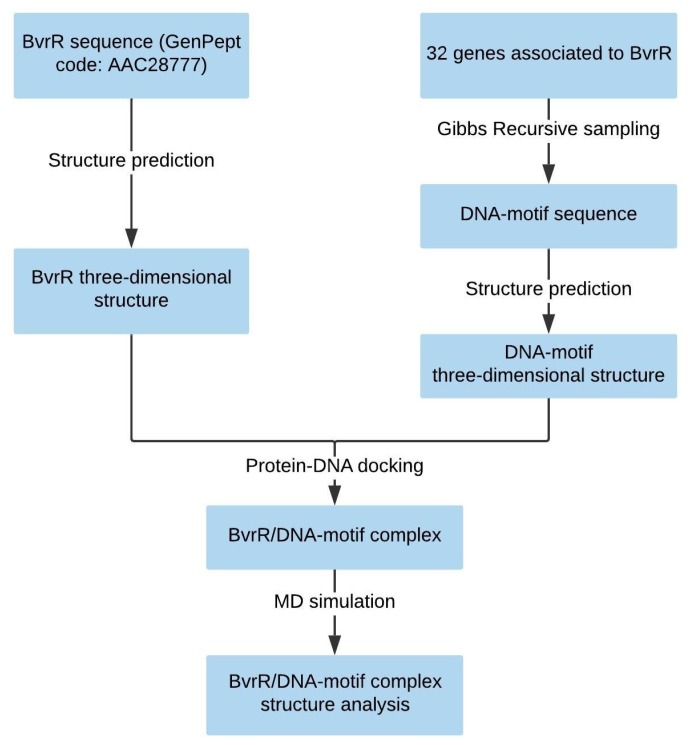
Work diagram of the prediction of BvrR/DNA motif interaction.

In this study, the structure of BvrR, the motif it recognizes in DNA, and the interaction between BvrR and DNA were predicted (Figure 1). In order to carry out this study, the three-dimensional structure of BvrR was constructed. Then the motif that is probably recognized by BvrR was predicted with Gibbs Recursive Sampling. The sequence AAHTGC (H represents the A, C, and T nucleotides) was found as the most probable motif recognized by BvrR. Therefore, the three-dimensional structure of the DNA-motif was constructed. Subsequently, the initial interaction between BvrR/DNA-motif was predicted by protein/DNA docking. Finally, the BvrR/DNA-motif interaction was analyzed by MD simulation.

## 2. Results and Discussion

### 2.1. Structure Prediction of BvrR

In the absence of homologous structures of significant similarity deposited in the Protein Data Bank (PDB), the tridimensional structure of the BvrR was predicted by means of a composite threading method using the I-TASSER software. The modeling yielded 5 structural models, all them with C-score values between −5 and 2. The best model was the only one with a positive C-score = +0.15, RMSD = 5.4 ± 3.4 Å, and TM-score = 0.73 ± 0.11 (Supplemental Information S1). According to Yang and Zang, the C-score estimates the confidence of the model with higher values meaning greater quality [24]. Moreover, models with C-score > −1.5 and TM-score > 0.5 have usually a correct fold. The TM-score is a measure of the structural similarity which is independent of the sequence length. TM-score values that are higher than 0.5 generally correspond to highly similar structures in the same SCOP/CATH fold family as reported by Xu and Zang [25]. Therefore we select only the optimal model as the best guess of the protein model.

**Figure 2:**
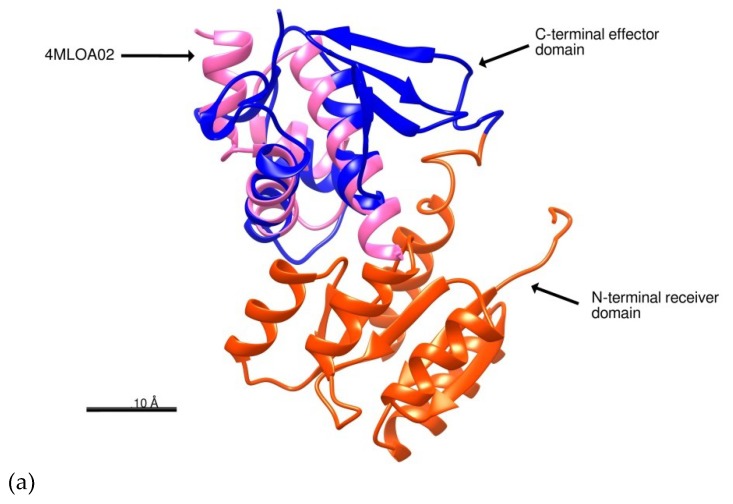

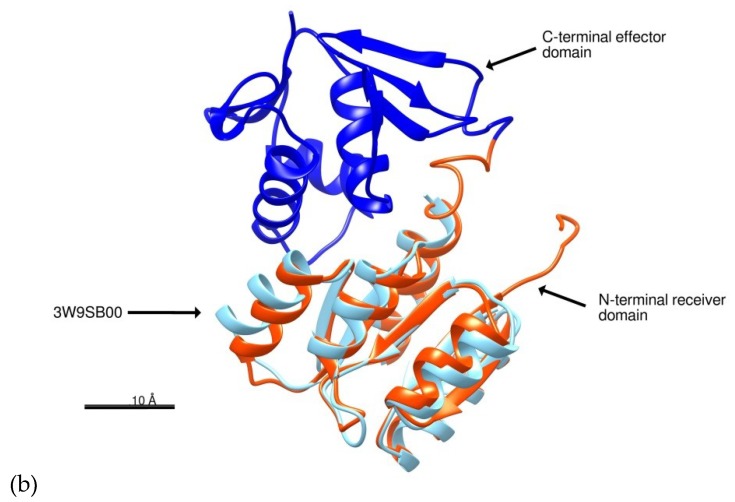
Structural prediction of BvrR. (**a**) *C*-terminal effector domain of BvrR (blue) compared with the domain 4MLOA02 of the transcriptional activator protein ToxT (pink). (**b**) *N*-terminal receiver domain of BvrR (red) compared with the domain 3W9SB00 of the *N*-terminal receiver domain of Response Regulator PmrA (light blue). The proteins were represented schematically with Chimera software developed by The National Institutes of Health (NIH) [31].

I-TASSER uses a Local Meta-Threading Server (LOMETS) to select templates with high significance in threading alignments to build the model; the significance is measured by the Z-score. An alignment with a Normalized Z-score > 1 indicates a good template for modeling. The best templates selected by LOMETS were the mutant of response regulator KdpE complexed to DNA (PDB code: 4kfcA), the response regulator KdpE complexed to its promoter (PDB code: 4knyA), and the response regulator MtrA (PDB code: 2gwrA) with normalized Z-scores of 4.21, 3.91, and 4.11, respectively (Supplemental information S1). We used the COACH server to predict possible ligand binding sites on BvrR. This site generates two binding site predictions using TM-SITE and S-SITE methods, which recognize ligand-binding templates from the BioLiP protein function database by binding-specific substructure and sequence profile comparisons. The server uses a confidence score (C-score) of predicted binding site ranking from 0 to 1, where a higher score indicates a more reliable prediction [26,27]. According to TM-SITE results, BvrR have binding sites for beryllium trifluoride ion, magnesium, and manganese with a C-score of 0.45; and accordingly to S-SITE, BvrR have binding sites for peptides, sulfate, and lanthanum ions with a C-score of 0.18 and two possible binding sites for nucleic acids with a C-score of 0.12 (Supplemental information S2, Figure S2.1 and S2.2). We also used COFACTOR server to predict possible BvrR biological functions. COFACTOR uses the three-dimensional (3D) structural model to thread through the BioLiP protein function database and identify Gene Ontology (GO), Enzyme Commission (EC), and functional insights. This server evaluates global and local similarity using the C-score^GO^, a confidence score of predicted GO terms, and values a range in between (0–1) where a higher value indicates a better prediction of the function [28,29]. COFACTOR defined the molecular function of BvrR in GO terms as a possible heterocyclic compound binding (GO:1901363, GO:0097159), a nucleic acid binding (GO:0003676), and a signal transducer activity (GO:0004871) with a Cscore^GO^ of 0.94, 0.89, and, 0.73 respectively. The biological process defined in GO terms showed a biological regulation (GO:0065007), signal transduction (GO:0007165), and a metabolic process activity (GO:0008152) with a Cscore^GO^ of 0.97, 0.89, and 0.71, respectively (Supplemental information S2). We also searched the evolutionary relationships of the BvrR domains in the CATH database. This resource identifies protein domain structures within 3D-protein structures from the PDB and assigns domains sharing evolutionary similarities to the same superfamily within the CATH hierarchical structure classification [30]. According to the sequence homology, the BvrR protein has two domains: an *N*-terminal receiver domain and a *C*-terminal effector domain. The *C*-terminal effector domain of BvrR has structural similarities with a domain of AraC-family transcriptional activator protein ToxT from *Vibrio cholerae* (PDB code: 4MLOA02) (Supplemental information S2, Figure 2a). This domain belongs to the Superfamily 3.40.50.12330 of the CATH classification, which is characterized for having protein histidine kinase activity (GO:0004673), phosphorelay response regulator activity (GO:0000156), pathogenesis (GO:0009405), and cellular response to osmotic stress (GO:0071470). The N-terminal receiver domain of BvrR shares a structural similarity with an N-terminal receiver domain of Response Regulator PmrA from *Escherichia coli* (PDB code: 3W9SB00) and an N-terminal receiver domain of a signal transduction histidine kinase from *Aspergillus oryzae* (PDB code: 3C97A01) (Supplemental information S2, Figure 2b). These domains belong to the Superfamily 3.40.50.2300 of the CATH classification, characterized by having protein binding activity (GO:0005515) and phosphorelay signal transduction system (GO:0000160). 

The quality of the predicted structure was first assessed using PROCHECK software, which evaluates the stereo-chemical data of the amino acids on the structure and compares them with the data obtained from a refined, high-resolution structure [32,33]. A Ramachandran plot was used to evaluate the BvrR model showing that 75.5% of the residues were in the most favored regions, 19.4% in the additional allowed regions, 2.3% in the generously allowed regions, and 2.8% in the disallowed regions (Supplemental Information S3). Although the software indicates that a good quality model would be expected to have over 90% in the most favored region, it also determines that unusual highlighted regions are not necessarily errors as such, but can be unusual features for which there is a reasonable explanation [32]. VERIFY3D software was used to determine the compatibility of the atomic model (3D) with its own amino acid sequence (1D) by assigning a structural class based on its location and environment [34,35]. Results showed that 85.77% of the residues have an averaged 3D–1D score ≥ 0.2, suggesting that the model is highly accurate (Supplemental Information S4). ERRAT software was used to analyze the BvrR model; it reliably identifies regions of error by examining the statistics of six types of non-covalently bonded atom-atom interactions (CC, CN, CO, NN, NO, and OO) in protein structures and it was used to evaluate the BvrR model [36]. In this analysis, a good protein structure was expected to show a confidence level above 95%. Results indicated that the structure has a quality factor of 96.53% (Supplemental Information S5). PROVE (PROtein Volume Evaluation) software was used to validate the volume-based structure of the BvrR model by determining the deviations of the atomic volumes from the standard values [37]. The program found 49 buried outliner protein atoms and an absolute Z score = 5.6 on the BvrR structure (Supplemental Information S6). Absolute Z-scores are used to identify problems in specific regions within a protein model. Proteins having absolute Z-scores > 3 occur at or near regions in the structure with unusual stereochemistry. This result correlates with the analysis obtained using PROCHECK with which we found unusual regions on the BvrR structure. Finally, ProSA (Protein Structure Analysis) software was used to validate the BvrR structure. This software calculates the overall model quality with the Z-score for a specific input structure, and the score is compared with those typically found for native proteins of similar size. If this score is outside a range characteristic for native proteins, the structure probably contains errors. The BvrR structure obtained a Z-score of −6.57, which is within the range of Z-scores found for native proteins of similar size, indicating that the overall quality of our model is high (Supplemental Information S7). 

### 2.2. Prediction of DNA-Motif Recognized by BvrR

**Figure 3:**
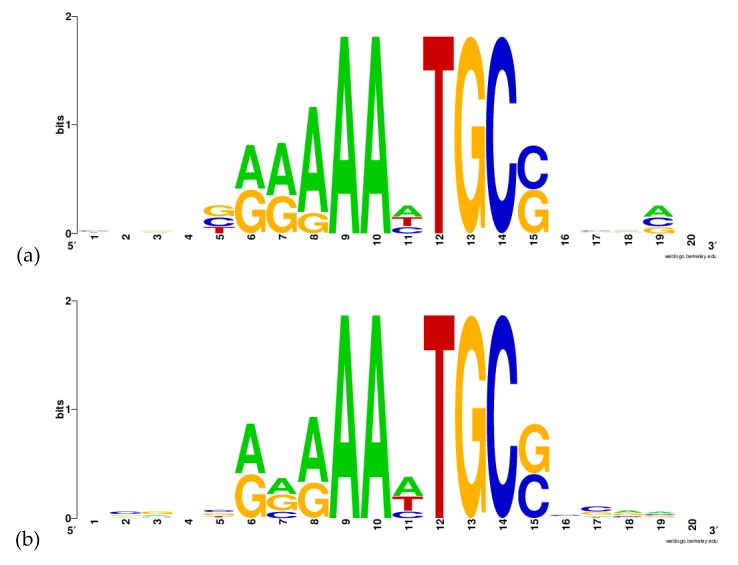

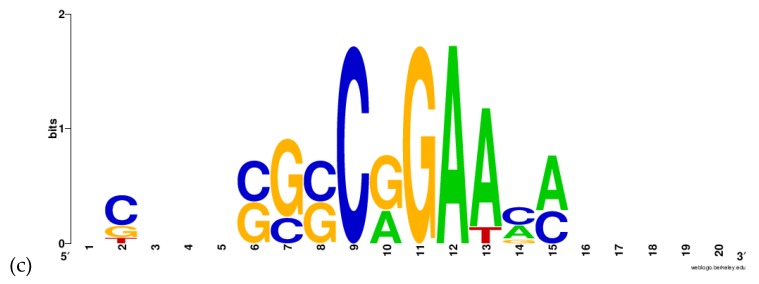
Sequences obtained with *Gibbs Recursive Sampling*. (**a**) The first motif was determined as the closest to the optimal alignment. (**b**) The second motif obtained was formed by events that occur with a probability greater than 50%. (**c**) The third motif obtained by the software was limited to three motifs per sequence and three different motifs in total. The schematic figures were created with WEBLOGO v 2.8.2.

Viadas et al. [18] performed a microarray analysis comparing the expression of all the RNA of a *B. abortus* wild-type and a mutant *bvrR*^−^ strain. The analysis showed that 127 genes had an alteration in their expression in the *bvrR*^−^ strain. Subsequently, real-time PCR analysis was performed to validate their data. They selected 48 genes related to carbon and nitrogen metabolism, control of external membrane proteins, transport, transcription factors, and virulence [18]. We selected and analyzed 32 genes of biological relevance from those analyzed in the real-time PCR and those mentioned in the discussion of the article to find the sequence of a potential DNA-motif recognized by BvrR. For the analysis, we used a 149 bp upstream and 21 bp downstream sequence from the initiation codon, making a final sequence of 170 bp (Supplemental information S8). Three motif analyses were performed with the Gibbs Motif Sampler software on the Gibbs Recursive Sampling mode. First, the software was configured to search for 10 base-pairs motifs, limited to one motif per sequence and two different motifs in all sequences. The first motif obtained was determined by the software as the most optimal result. The data of this motif, its location and the genes where this sequence was found are shown in Table S1 (Supplemental information S9) while the graphic representation of this sequence is shown in Figure 3a. The second motif obtained was determined as being formed by hits that occur with a probability greater than 50% in an optimal alignment in 500 iterations. Table S2 shows the data from this motif (Supplemental information S9) and the graphic representation of this sequence is shown in Figure 3b. On the third motif analysis, the software was configured to search for 10 bp motifs, limited to three motifs per sequence and three different motifs in all sequences. The data of this motif, its location, and the genes where this sequence was found are shown in Table S3 (Supplemental information S9). Finally, the graphic representation of this sequence is shown in Figure 3c.

The three-dimensional structure of the DNA-motif AAHTGC (H represents A, C, and T nucleotides in FASTA format) was built using the 3D-DART server. To fill the gap in the sequence and make the DNA sequence longer to evaluate the docking with BvrR, we choose the sequence showing the motif for the *omp25a* gene, which codes for an Omp25 protein (Supplemental Information S9). The final sequence model built as double-stranded DNA-B was GCGGCACG**AAATGC**CCCATT.

### 2.3. Docking of BvrR into the DNA-motif

**Figure 4:**
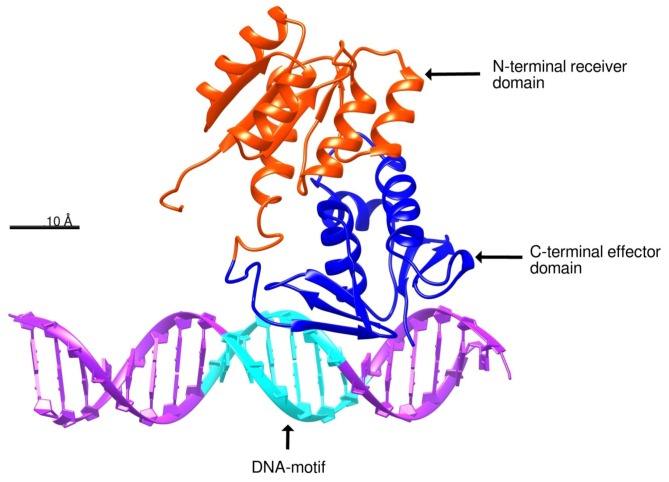
Docking prediction between BvrR and DNA. The docked pose showed the interaction between the *C*-terminal effector domain of BvrR (blue) and the DNA-motif (cyan). The docking analysis was performed with HDOCK software. The protein was represented schematically with Chimera software developed by The National Institutes of Health (NIH) [31].

The docking analysis was performed with HDOCK server, developed by Huang Laboratory at the Huazhong University of Science & Technology [38,39]. According to structural similarity, the docked pose 7 showed a probable interaction between the DNA-motif and the C-terminal effector domain of BvrR that shares structure similarity with a domain of the transcription regulator protein ToxT from *Vibrio cholerae* (PDB code: 4MLOA). This docked pose obtained a Docking score of −448.3 and ligand RMSD = 126.84 Å (Figure 4) (Supplemental information S10). HDOCK uses a distance-dependent knowledge-based scoring function (ITScore-PP) to predict interactions. The ITScore-PP improves the interatomic pair potentials using a statistical mechanics-based iterative method, in which the pairwise distance-dependent atomic interaction potentials were derived from experimentally determined complex structures [40,41,42]. 

### 2.4. MD Simulations

**Figure 5:**
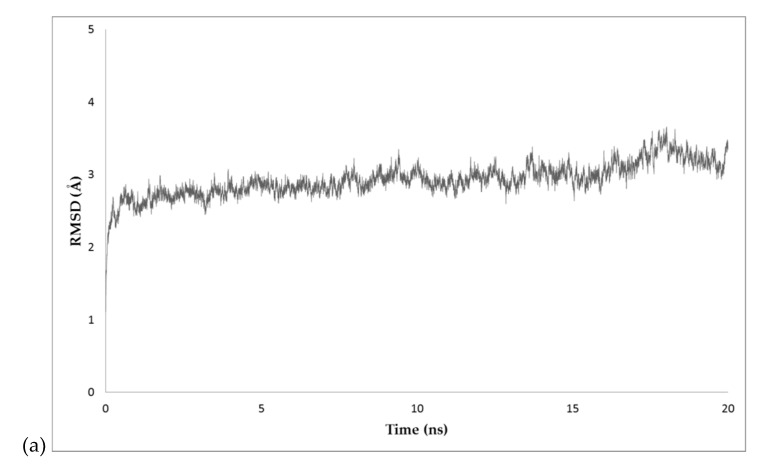

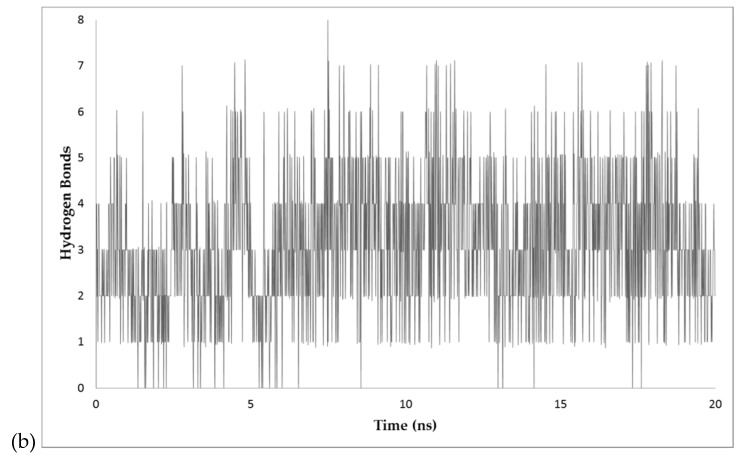
Predicted interactions during EMD simulation. (**a**) RMSD of BvrR protein docked to DNA by *C*-terminal effector domain with respect to the initial structure during 20 ns simulation. (**b**) Intermolecular hydrogen bonds between the C-terminal domain and DNA during 20 ns simulation. Data were obtained and analysis was performed with VMD software [43].

The docked pose 7 was selected to carry out the Equilibrium Molecular Dynamics (EMD) simulation (Supplemental Information S11). The RMSD (root mean square deviation) was plotted during the 20 ns simulation. The RMSD values were plotted and are shown in Figure 5a. Additionally, the number of hydrogen bonds between BvrR and the DNA-motif during the EMD simulation were plotted. The average number of hydrogen bonds was 3.13 (Figure 5b) (Supplemental Information S12). 

**Figure 6:**
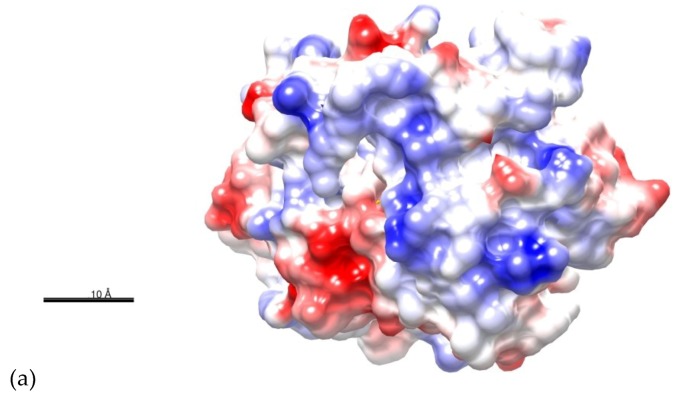

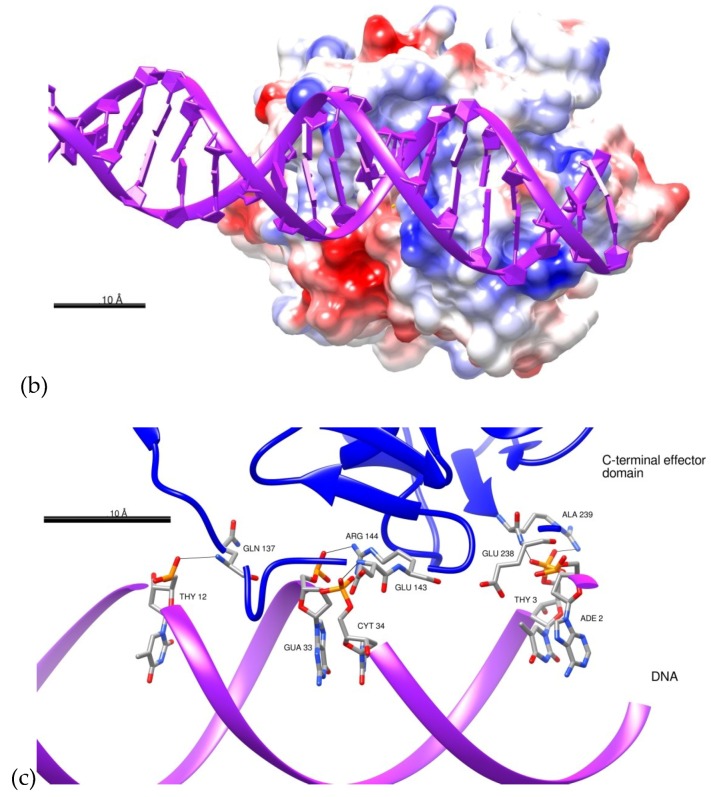
Predicted interactions between the *C*-terminal effector domain of BvrR and DNA. (**a**) Coulombic surface coloring of the *C*-terminal effector domain. The areas in royal blue represent surface where energy is 10 Kcal/mol; areas of intense red represents surface where the energy is −10 Kcal/mol, and white areas represent surface where energy is 0 Kcal/mol. (**b**) Coulombic surface coloring of *C*-terminal effector domain with DNA. (**c**) Intermolecular interactions between *C*-terminal effector domain (blue) and DNA (purple); HBs are indicated by black lines (Frame 7507). Proteins were represented schematically with Chimera software developed by The National Institutes of Health (NIH) [31].

We analyzed the Coulombic surface force of the *C*-terminal domain on frame 7507, to identify the charge in the surface of the *C*-terminal effector domain (Figure 6a,b). To analyze the energies involved in the *C*-terminal effector domain and the DNA, two frames (Frames 5523 and 7507) were extracted from the MD simulation and analyzed with the FireDock server. Averaging the results of both frames, the docked pose 7 obtained an attractive Van der Waals (VdW) force of −26.77 Kcal/mol, a repulsive VdW force of 7.56 Kcal/mol, an atomic contact energy (ACE) of 5.18 Kcal/mol, a global binding energy of −16.31 Kcal/mol and five possible hydrogen bonds between the BvrR protein and DNA (Figure 6c). To evaluate the energies involved in the DNA when it has a different motif, we analyzed frame 7507 interacting with the same DNA but changed the motif sequence AAHTGC for CCGGTA. This docked pose obtained an attractive VdW force of −15.78 Kcal/mol, a repulsive VdW force of 6.16 Kcal/mol, an ACE of 3.58 Kcal/mol, Global binding energy of −2.91 Kcal/mol, and one possible hydrogen bond (Supplemental Information S13). 

## 3. Materials and Methods

### 3.1. Structure Prediction

The BvrR sequence was obtained from the Gene Products Data Bank (GenPept code AAC28777) [44]. The analysis and modeling of BvrR were performed using I-TASSER (Iterative Threading ASSEmbly Refinement) Protein Structure & Function Predictions developed by Zhang Lab University of Michigan [45,46,47] to predict the possible three-dimensional structure of the BvrR protein. Molecular graphics and analyses were performed with UCSF Chimera, developed by the Resource for Biocomputing, Visualization, and Informatics at the University of California, San Francisco, with support from NIH P41-GM103311 [31]. The DNA three-dimensional structure modeling was performed using the 3DNA-Driven DNA Analysis and Rebuilding Tool server (3D-DART server) [48]. The quality analysis of the predicted structure was conducted using SAVES (PROCHECK, VERIFY3D, ERRAT, PROVE) and ProSA servers. Finally, the modeled structure was visualized using Chimera v 1.13.

### 3.2. Gibbs Sampling

The analysis of the possible regulator zone on the DNA of BvrR was made with Gibbs Motif Sampler Homepage, *Gibbs* v 3.1 [49,50,51]. We used 32 genes from a previous work for the analysis [18] and selected 170 bp upstream of the starting codon of each gene, where the regulator zone is probably located (Supplemental information S8). The Recursive Sampling mode was specifically used to perform three motif analyses. Firstly, we searched for 10-bp motifs, one motif per sequence, and a maximum of 2 motifs on all the sequences. Afterward, we searched for 10-bp motifs, 3 motifs per sequence, and a maximum of 3 motifs on all the sequences. To graphically represent the multiple sequence alignment, we used WebLogo v 2.8.2 [52].

### 3.3. Protein-DNA Docking

Protein-DNA docking was performed to analyze the interactions between BvrR and the DNA sequence found with Gibbs sampling. The analysis was carried out with HDOCK server, developed by Huang Laboratory at the Huazhong University of Science & Technology [38,39,40,41]. HDOCK server can model protein–protein and protein–DNA/RNA docking based on a hybrid docking algorithm of template-based modeling and free docking, in which cases with misleading templates can be rescued by the free docking protocol [39].

### 3.4. Molecular Dynamics Simulations

The docking structures in PDB format were refined with MolProbity server developed by the Department of Biochemistry at Duke University [53]. The Equilibrium Molecular dynamics simulations of the complex were performed with Nanoscale Molecular Dynamics (NAMD2) version 2.12. a molecular dynamics code based on Charm++ parallel objects, designed for high-performance simulation of large biomolecular systems [54]. Additionally, Visual Molecular Dynamics (VMD) version 1.9 was used for simulation setup and trajectory analysis [43,55,56]. The PSF and PDB model representations were build using the CHARMM36 Force Field with the complex immersed into a rectangular periodic box of water of dimensions 60 Å x 92 Å x 90Å; the distance established from the molecule to the edge of the box was 10 Å. The system was neutralized with NaCl. The dynamics ran to a constant temperature of 310 K in explicit solvent. The simulations of the system were carried out using the Langevin dynamics [57], consisting of minimization and equilibration of the molecule in the water box, with a timestep of 2 fs/step considering as rigid all bonds. A thousand steps for minimization were employed and a total number of 107 of simulation steps for a total simulated time of 20 ns for the production dynamics [54]. 

### 3.5. Analysis of Dynamics Simulations

First, we used the output files from the minimization and equilibration of the molecule to calculate the RMSD values and analyze the extent of equilibration of the simulation; the RMSD values were calculated for the entire molecule (excluding the hydrogen) every 2 fs of the simulation. With this information, we constructed a plot of RMSD over time to know the stability of the protein. If the RMSD is stable at the end of the run, it means that the molecule is equilibrated. To analyze the binding energies we used the FireDock server developed by the Raymond and Beverly Sackler Faculty of Exact Sciences at the Tel Aviv University [58]. The FireDock server scores the interaction between the protein and the DNA according to Atomic Contact Energy [59], softened Van der Waals interactions, partial electrostatics, and additional estimations of the binding free energy. 

## 4. Discussion

In the structure prediction, the evolutionary relationships of the BvrR domains showed that the *C*-terminal effector domain of BvrR has high homology with response regulators as proteins KdpE (4knyA), MtrA (2gwrA), and ToxT (4MLOA). This domain belongs to the Superfamily 3.40.50.12330 which have known histidine kinase and phosphorelay response regulator activities related to pathogenesis and response to osmotic stress. The *N*-terminal receiver domain has high homology with protein PmrA (3W9SB) and a signal transduction histidine kinase (3C97A). This domain belongs to the Superfamily 3.40.50.2300 and is the one that interacts with the sensor BvrS, which correlates with its protein binding activity and phosphorelay signal transduction activities. All these results, including the ones obtained with VERIFY3D, ERRAT and ProSA that prove the accuracy of the model, validate the BvrR structural model, even though some small regions were modeled in an unusual way (accordingly to PROCHECK and PROVE). This may be because the modeling template contained those variations, which were transferred to the model; it may also be due to the lack of better templates on which to base the prediction of the BvrR structure.

In the search of the DNA motif recognized by BvrR, it can be observed that the first two motifs found by the software have the same sequence, AAHTGC. This sequence was found before the coding sequence for 13 genes, as nitric oxide reductase NorC, Lipoprotein, methyltransferase Cfa, transcriptional regulator OmpR, GntR regulator, thermal shock protein HtpX, and glutaminase R, among others (Supplemental Information S9). Most importantly, the motif was found for the outer membrane protein Omp25, which is known to be regulated by BvrR [16]. This could indicate that the motif is probably the one recognized by this regulator on the DNA. The third motif was found only on 3 genes; for this reason, this motif was discarded for further analysis. Interestingly, the sequence AAHTGC has a great similarity to the sequence AAATGTG, one of the sequences recognized by the response regulator KdpE (PDB code: 4knyA). The latter was one of the threading templates used by I-TASSER to build the BvrR model, as mentioned above, and was the only template crystalized with its DNA motif. It should be noted that the motif AAHTGC was found after the start codon in the genes coding for a Heat Shock Protein HtpX (BAB1_1821) and in the Negative exopolysaccharide regulator ExoR (BAB1_0891). This suggested a possible negative regulation on those genes (Table S4, Supplemental Information S9).

In the docking analysis, we selected the docked pose 7 because it showed an interaction between BvrR and the DNA-motif related to the one predicted by COACH server with the method S-SITE (Figure S2.1, Supplemental Information S2) and because it also had a good docking score, showing a high probability that it is the right zone of interaction. This was related to the EMD simulation, where the RMSD values were kept constant almost from the beginning and the number of hydrogen bonds remained stable, indicating that the interaction between the *C*-terminal effector domain and the DNA-motif is in equilibrium. This also correlated with the analysis of the Coulombic surface force on the site of interaction, where the *C*-terminal effector domain was positively charged and interacted with the negatively charged DNA, making the union in that area highly probable. Finally, those results were confirmed with the analysis of the energies involved in the interaction, where the global binding energy between BvrR and the AAHTGC motif was higher compared to that observed when using an unrelated DNA-motif. 

All results shown above were performed by computational models, and need to be validated experimentally by means of ChIP-Seq and the crystallization of BvrR interacting with DNA. Unfortunately, specific monoclonal antibodies against BvrR, necessary to purify the protein and carry out these studies, are not available. In the future, we would like to continue the research on this response regulator, as it is possibly a major virulence factor in the infection caused by *Brucella*. We believe that knowledge of the structure, binding site to DNA, and regulated genes could lead to a better understanding of the infection mechanism, and that knowledge in the future could aid in the selection of new targets for the development of new vaccines. Also, BvrR could be used as a new biomarker for diagnostic chronic infections of brucellosis, as Vitale et al. [60] used the cyclin-dependent kinase inhibitor p16^INK4a^, in high-risk HPV infections, to identify which low-grade intraepithelial lesion (LSIL) cases were inclined to the progression of the disease and the possible development of cervical cancer.

## 5. Conclusions

According to the results obtained, the three-dimensional structure of BvrR was consistently predicted. It was also found that the AAHTGC sequence is likely the motif recognized in the DNA by BvrR. The BvrR protein will probably interact with the DNA motif sequence found in Gibbs Recursive Sampler. The interaction between the DNA and the *C*-terminal effector domain showed a good equilibrium when analyzing the RMSD of all atoms as compared to the initial position of the molecule. The hydrogen bonds between BvrR and DNA remained stable during the simulation. According to the analysis of the surface electrical charge, the BvrR *C*-terminal effector domain is positively charged in the area where it interacts with the DNA, which possibly favors that interaction. When analyzing the energies involved, high attractive VdW energies and global binding energy were observed. Likewise, the analysis showed little interference of repulsive VdW energies as compared to the observed energies when the DNA-motif is exchanged for an unrelated one. The data obtained in this work were in silico and must be validated experimentally in the future.

## Supplementary Materials

The following are available online. Supplemental Information S1: I-TASSER results for predicted BvrR structure; Supplemental Information S2: CATH results for BvrR homology; Supplemental Information S3: PROCHECK results for BvrR; Supplemental Information S4: Verify 3D results for BvrR; Supplemental Information S5: ERRAT results for BvrR; Supplemental Information S6: PROVE results for BvrR; Supplemental Information S7: ProSA results for BvrR; Supplemental Information S8: Sequences of the 32 genes used for the Gibbs Sampling analysis; Supplemental Information S9: Gibbs recursive sampling results obtained with the 32 sequences; Supplemental Information S10: HDOCK docking results for BvrR and DNA-motif; Supplemental Information S11: MD simulations results for docked pose 7; and Supplemental Information S12: Firedock analysis results for frames 5523 and 7507.
