# Supplementary material for: Prediction of Structure and Molecular Interaction with DNA of BvrR, a Virulence-Associated Regulatory Protein of Brucella
Source: Molecules. 2019 Aug 29;24(17):3137. doi: 10.3390/molecules24173137 (PMC6749498; doi:10.3390/molecules24173137)
Supplement: Supplementary file 1 [file molecules-24-03137-s001.zip › molecules-545148-revised-1-SM - final/Supplemental Information S10/Supplemental Information S10.pdf]

Supplemental Information S10

HDOCK results for BvrR docked to DNA

Your HDOCK results for job *BvrR*

NOTE: No template was used for the **Ligand** Molecule!

You may **bookmark this page** and come back later to view the results.

Template Information

Molecule	PDB ID	Chain ID	Align_length	Coverage	Seq_ID (%)
Receptor	4B09	A	230	0.962	37.8
Ligand					

Summary of the top 10 models

Rank	1	2	3	4	5	6	7	8	9	10
**Docking Score**	-485.79	-484.78	-481.22	-458.31	-453.66	-450.74	-448.30	-447.17	-446.14	-440.93
**Ligand rmsd (Å)**	108.93	142.58	91.94	89.52	144.00	90.35	126.84	91.31	138.27	128.13

(a) Row 1: The ranks of the models.
(b) Row 2: The docking energy scores.
(c) Row 3: The ligand RMSDs from the input structures or modeled structures by homology modeling.
