# Supplementary material for: Prediction of Structure and Molecular Interaction with DNA of BvrR, a Virulence-Associated Regulatory Protein of Brucella
Source: Molecules. 2019 Aug 29;24(17):3137. doi: 10.3390/molecules24173137 (PMC6749498; doi:10.3390/molecules24173137)
Supplement: Supplementary file 1 [file molecules-24-03137-s001.zip › molecules-545148-revised-1-SM - final/Supplemental Information S13/Supplemental Information S13.pdf]

Supplemental Information S13

FireDock results of BvrR interacting with DNA

Frame 5523

Global Energy (Kcal/mol)	Attractive VdW (Kcal/mol)	Repulsive VdW (Kcal/mol)	ACE (Kcal/mol)
-15.44	-30.65	9.58	7.66

Frame 7507

Global Energy (Kcal/mol)	Attractive VdW (Kcal/mol)	Repulsive VdW (Kcal/mol)	ACE (Kcal/mol)
-17.19	-22.90	5.55	2.71

BvrR with DNA with different motif sequence

Global Energy (Kcal/mol)	Attractive VdW (Kcal/mol)	Repulsive VdW (Kcal/mol)	ACE (Kcal/mol)
-2.91	-15.78	6.16	3.58
