# Supplementary material for: Prediction of Structure and Molecular Interaction with DNA of BvrR, a Virulence-Associated Regulatory Protein of Brucella
Source: Molecules. 2019 Aug 29;24(17):3137. doi: 10.3390/molecules24173137 (PMC6749498; doi:10.3390/molecules24173137)
Supplement: Supplementary file 1 [file molecules-24-03137-s001.zip › molecules-545148-revised-1-SM - final/Supplemental Information S1/Supplemental Information S1.pdf]

[[Home](#)] [[Server](#)] [[Queue](#)] [[About](#)] [[Remove](#)] [[Statistics](#)]

I-TASSER results for job id S425338

(Click on S425338_results.tar.bz2 to download the tarball file including all modeling results listed on this page. Click on Annotation of I-TASSER Output to read the instructions for how to interpret the results on this page. Model results are kept on the server for 60 days, there is no way to retrieve the modeling data older than 2 months)

Submitted Sequence in FASTA format

```
>protein
MKEASATQTIALVDDDRNILTSVSIALESEGYRVETYTDGASALDGLMARPPNLAIFDIK
MPRMDGMELLRRLRQKSDLPVIFLTSKDDEIDELFGLKMGADDFITKPFSQRLLVERVKA
VLRRVAARDGTAKPAGQQAKSLERGQLVMDQERHTCTWKGEPVTLTVTEFLILHSLAQRP
GVVKSRDALMDAAYDEQVYVDDRTIDSHIKRLRKKFKAVDDSFEMIETLYGVGYRFEA
```

Predicted Secondary Structure

```
                   20                  40                  60                  80                  100                 120
                    |                   |                   |                   |                   |                   |
Sequence   MKEASATQTIALVDDDRNILTSVSIALESEGYRVETYTDGASALDGLMARPPNLAIFDIKMPRMDGMELLRRLRQKSDLPVIFLTSKDDEIDELFGLKMGADDFITKPFSQRLLVERVKAVLRRVAAR
Prediction CCCCCCCCCSSSSCCCHHHHHHHHHHHHHCCCSSSSSCCHHHHHHHHHHCCCCSSSSSCCCCCCCHHHHHHHHHHCCCCCSSSSSSCCCHHHSSCCCCCCCCSSSSCCCCHHHHHHHHHHHHCCCCCC
Conf.Score 986678885899879889999999999997798899978989999998726999999936789999999999997118998899983764754313566655876238999999999999998118644
           H:Helix; S:Strand; C:Coil
```

Predicted Solvent Accessibility

```
                   20                  40                  60                  80                  100                 120
                    |                   |                   |                   |                   |                   |
Sequence   MKEASATQTIALVDDDRNILTSVSIALESEGYRVETYTDGASALDGLMARPPNLAIFDIKMPRMDGMELLRRLRQKSDLPVIFLTSKDDEIDELFGLKMGADDFITKPFSQRLLVERVKAVLRRVAAR
Prediction 866544543000011264004003300442313123022064004304745010000002027320240033017537000000000344430202103020201001232331010102010102344
           Values range from 0 (buried residue) to 9 (highly exposed residue)
```

Predicted normalized B-factor

(B-factor is a value to indicate the extent of the inherent thermal mobility of residues/atoms in proteins. In I-TASSER, this value is deduced from threading template proteins from the PDB in combination with the sequence profiles derived from sequence databases. The reported B-factor profile in the figure below corresponds to the normalized B-factor of the target protein, defined by B=(B'-u)/s, where B' is the raw B-factor value, u and s are respectively the mean and standard deviation of the raw B-factors along the sequence. Click here to read more about predicted normalized B-factor)

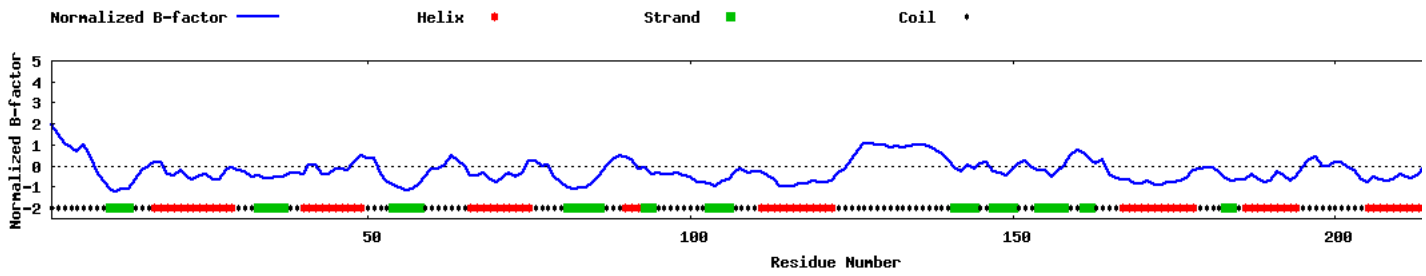

Top 10 threading templates used by I-TASSER

(I-TASSER modeling starts from the structure templates identified by LOMETS from the PDB library. LOMETS is a meta-server threading approach containing multiple threading programs, where each threading program can generate tens of thousands of template alignments. I-TASSER only uses the templates of the highest significance in the threading alignments, the significance of which are measured by the Z-score, i.e. the difference between the raw and average scores in the unit of standard deviation. The templates in this section are the 10 best templates selected from the LOMETS threading programs. Usually, one template of the highest Z-score is selected from each threading program, where the threading programs are sorted by the average performance in the large-scale benchmark test experiments.)

Rank	PDB Hit	Iden1	Iden2	Cov	Norm. Z-score	Download Align.	20 | 40 | 60 | 80 | 100 |
							Sec.Str CCCCCCCCCSSSSCCCHHHHHHHHHHHHHCCCSSSSSCCHHHHHHHHHHCCCCSSSSSCCCCCCCHHHHHHHHHHCCCCCSSSSSSCCCHHHSSCCCCCCC<br>Seq MKEASATQTIALVDDDRNILTSVSIALESEGYRVETYTDGASALDGLMARPPNLAIFDIKMPRMDGMELLRRLRQKSDLPVIFLTSKDDEIDELFGLKMGA
1	4kfcA	0.29	0.30	0.93	4.21	Download	M------ANVLIVEDEQAIRRFLRTALEGDGMRVFEAETLQRGLLEAATRKPDLIILDLGLPDGDGIEFIRDLRQWSAVPVIVLSARSEESDKIAALDAGA<br>HSAPDPLVKFSDVTVDLAARVIHRGEEEVHLTPIEFRLLAVLLNNAGKVLTQRQLLNQVWGPNAVEHSHYLRIYMGHLRQKLEQDPARPRHFITATGIGYR
2	4knyA	0.29	0.29	0.94	3.91	Download	------MANVLIVEDEQAIRRFLRTALEGDGMRVFEAETLQRGLLEAATRKPDLIILDLGLPDGDGIEFIRDLRQWSAVPVIVLSARSEESDKIAALDAGA<br>-
3	2gwrA	0.35	0.34	0.94	4.11	Download	---DTMRQRILVVDDDASLAEMLTIVLRGEGFDTAVIGDGTQALTAVRELRPDLVLLDLMLPGMNGIDVCRVLRADSGVPIVMLTAKTDTVDVVLGLESGA<br>ADTRLVNVHVQRLRAKVEKDPENPTVVLTVRGVGYKAGPP
4	2oqr	0.35	0.33	0.95	1.85	Download	------ATSVLIVEDEESLADPLAFLLRKEGFEATVVTDGPAALAEFDRAGADIVLLDLMLPGMSGTDVCKQLRARSSVPVIMVTARDSEIDKVVGLELGA<br>-
5	5u8k	0.27	0.26	0.92	1.39	Download	------GKRILLLEKERNLAHFLSLELQKEQYRVDLVEEGQKALSALQ-TDYDLILLNVNL--GDAQDFAEKLSRTKPASVIILDHWEDLQEELEVVQRFA<br>VKGQKSYIKTVRGVGYTQEG-
6	4kfcA	0.29	0.30	0.93	3.87	Download	------MANVLIVEDEQAIRRFLRTALEGDGMRVFEAETLQRGLLEAATRKPDLIILDLGLPDGDGIEFIRDLRQWSAVPVIVLSARSEESDKIAALDAGA<br>APDPLVKFSDVTVDLAARVIHRGEEEVHLTPIEFRLLAVLLNNAGKVLTQRQLLNQVWGPNAVEHSHYLRIYMGHLRQKLEQDPARPRHFITATGIGYRFM
7	5u8k	0.27	0.26	0.92	2.06	Download	------GKRILLLEKERNLAHFLSLELQKEQYRVDLVEEGQKALSALQ-TDYDLILLNVNL--GDAQDFAEKLSRTKPASVIILDHWEDLQEELEVVQRFA

							VKGQKSYIKTVRGVGYTQEG-
8	2gwrA	0.35	0.34	0.94	5.86	Download	---DTMRQRILVVDDDASLAEMLTIVLRGEGFDTAVIGDGTQALTAVRELRPDLVLLDLMLPGMNGIDVCRVLRADSGVPIVMLTAKTDTVDVVLGLESGA[illegible] ADTRLVNVHVQRLRAKVEKDPENPTVVLTVRGVGYKAGPP
9	2oqrA	0.34	0.33	0.95	4.02	Download	------ATSVLIVEDEESLADPLAFLLRKEGFEATVVTDGPAALAEFDRAGADIVLLDLMLPGMSGTDVCKQLRARSSVPVIMVTARDSEIDKVVGLELGA[illegible] -
10	2oqrA	0.34	0.33	0.95	4.94	Download	------ATSVLIVEDEESLADPLAFLLRKEGFEATVVTDGPAALAEFDRAGADIVLLDLMLPGMSGTDVCKQLRARSSVPVIMVTARDSEIDKVVGLELGA[illegible] -

(a) All the residues are colored in black; however, those residues in template which are identical to the residue in the query sequence are highlighted in color. Coloring scheme is based on the property of amino acids, where polar are brightly coloured while non-polar residues are colored in dark shade. (more about the colors used)
(b) Rank of templates represents the top ten threading templates used by I-TASSER.
(c) Ident1 is the percentage sequence identity of the templates in the threading aligned region with the query sequence.
(d) Ident2 is the percentage sequence identity of the whole template chains with query sequence.
(e) Cov represents the coverage of the threading alignment and is equal to the number of aligned residues divided by the length of query protein.
(f) Norm. Z-score is the normalized Z-score of the threading alignments. Alignment with a Normalized Z-score >1 mean a good alignment and vice versa.
(g) Download Align. provides the 3D structure of the aligned regions of the threading templates.
(h) The top 10 alignments reported above (in order of their ranking) are from the following threading programs:
1: MUSTER 2: FFAS-3D 3: SPARKS-X 4: HHSEARCH2 5: HHSEARCH I 6: Neff-PPAS 7: HHSEARCH 8: pGenTHREADER 9: wdPPAS 10: PROSPECT2

Top 5 final models predicted by I-TASSER

(For each target, I-TASSER simulations generate a large ensemble of structural conformations, called decoys. To select the final models, I-TASSER uses the SPICKER program to cluster all the decoys based on the pair-wise structure similarity, and reports up to five models which corresponds to the five largest structure clusters. The confidence of each model is quantitatively measured by C-score that is calculated based on the significance of threading template alignments and the convergence parameters of the structure assembly simulations. C-score is typically in the range of [-5, 2], where a C-score of a higher value signifies a model with a higher confidence and vice-versa. TM-score and RMSD are estimated based on C-score and protein length following the correlation observed between these qualities. Since the top 5 models are ranked by the cluster size, it is possible that the lower-rank models have a higher C-score in rare cases. Although the first model has a better quality in most cases, it is also possible that the lower-rank models have a better quality than the higher-rank models as seen in our benchmark tests. If the I-TASSER simulations converge, it is possible to have less than 5 clusters generated; this is usually an indication that the models have a good quality because of the converged simulations.)

- More about C-score
- Local structure accuracy profile of the top five models

(By right-click on the images, you can export image file or change the configurations, e.g. modifying the background color or stopping the spin of your models)

- Download Model 1
- C-score=0.15 (Read more about C-score)
- Estimated TM-score = 0.73±0.11
- Estimated RMSD = 5.4±3.4Å

- Download Model 2
- C-score = -1.32

- Download Model 3
- C-score = -1.43

Proteins structurally close to the target in the PDB (as identified by TM-align)

(After the structure assembly simulation, I-TASSER uses the TM-align structural alignment program to match the first I-TASSER model to all structures in the PDB library. This section reports the top 10 proteins from the PDB that have the closest structural similarity, i.e. the highest TM-score, to the predicted I-TASSER model. Due to the structural similarity, these proteins often have similar function to the target. However, users are encouraged to use the data in the next section 'Predicted function using COACH' to infer the function of the target protein, since COACH has been extensively trained to derive biological functions from multi-source of sequence and structure features which has on average a higher accuracy than the function annotations derived only from the global structure comparison.)

Top 10 Identified stuctural analogs in PDB

Click to view	Rank	PDB Hit	TM-score	RMSD[a]	IDEN[a]	Cov	Alignment
	1	2gwrA	0.857	1.58	0.353	0.912	Download
	2	1ys7B	0.769	2.75	0.324	0.904	Download
	3	5u8kA	0.769	2.79	0.245	0.920	Download
	4	3q9sA	0.640	3.33	0.275	0.791	Download
	5	2v0nA	0.612	4.06	0.223	0.766	Download
	6	1s8nA	0.554	4.02	0.182	0.699	Download
	7	1rnlA	0.552	3.36	0.206	0.674	Download
	8	1a2oA	0.551	4.40	0.162	0.766	Download
	9	4gvpA	0.548	2.40	0.189	0.611	Download
	10	5m7nA	0.548	3.52	0.174	0.648	Download

(a) Query structure is shown in cartoon, while the structural analog is displayed using backbone trace.
(b) Ranking of proteins is based on TM-score of the structural alignment between the query structure and known structures in the PDB library.
(c) RMSD[a] is the RMSD between residues that are structurally aligned by TM-align.
(d) IDEN[a] is the percentage sequence identity in the structurally aligned region.
(e) Cov represents the coverage of the alignment by TM-align and is equal to the number of structurally aligned residues divided by length of the query protein.

Predicted function using COFACTOR and COACH

(This section reports biological annotations of the target protein by COFACTOR and COACH based on the I-TASSER structure prediction. While COFACTOR deduces protein functions (ligand-binding sites, EC and GO) using structure comparison and protein-protein networks, COACH is a meta-server approach that combines multiple function annotation results (on ligand-binding sites) from the COFACTOR, TM-SITE and S-SITE programs.)

Ligand binding sites

Click to view	Rank	C-score	Cluster size	PDB Hit	Lig Name	Download Complex	Ligand Binding Site Residues
	1	0.42	20	4h60A	CA	Rep, Mult	14,15,58,60,61,107

2	0.10	5	4nicC	BEF	Rep, Mult	15,58,60,85,86,107
3	0.04	3	1zdmA	XE	Rep, Mult	13,14,23,56,57,58,84
4	0.02	1	1YS7B	1YS7B00	Rep, Mult	90,93,104,201,203
5	0.02	1	1YS6B	1YS6B00	Rep, Mult	163,164,165,215,216

Download the residue-specific ligand binding probability, which is estimated by SVM.
Download the all possible binding ligands and detailed prediction summary.
Download the templates clustering results.

(a) **C-score** is the confidence score of the prediction. C-score ranges [0-1], where a higher score indicates a more reliable prediction.
(b) **Cluster size** is the total number of templates in a cluster.
(c) **Lig Name** is name of possible binding ligand. Click the name to view its information in the BioLiP database.
(d) **Rep** is a single complex structure with the most representative ligand in the cluster, i.e., the one listed in the **Lig Name** column.
Mult is the complex structures with all potential binding ligands in the cluster.

Enzyme Commission (EC) numbers and active sites

Click to view	Rank	Cscore^{EC}	PDB Hit	TM-score	RMSD^{a}	IDEN^{a}	Cov	EC Number	Active Site Residues
	1	0.258	2v0nA	0.612	4.06	0.223	0.766	2.7.7.65	117,122
	2	0.247	1a2oA	0.551	4.40	0.162	0.766	3.1.1.61	NA
	3	0.242	1fuiA	0.535	4.68	0.081	0.782	5.3.1.25	45
	4	0.233	2p4eA	0.540	4.44	0.080	0.761	3.4.21.-	NA
	5	0.232	3bpsA	0.535	4.62	0.086	0.774	3.4.21.-	NA

Click on the radio buttons to visualize predicted active site residues.

(a) Cscore^{EC} is the confidence score for the EC number prediction. Cscore^{EC} values range in between [0-1]; where a higher score indicates a more reliable EC number prediction.
(b) TM-score is a measure of global structural similarity between query and template protein.
(c) RMSD^{a} is the RMSD between residues that are structurally aligned by TM-align.
(d) IDEN^{a} is the percentage sequence identity in the structurally aligned region.
(e) Cov represents the coverage of global structural alignment and is equal to the number of structurally aligned residues divided by length of the query protein.

Gene Ontology (GO) terms

Top 10 homologous GO templates in PDB

Rank	Cscore^{GO}	TM-score	RMSD^{a}	IDEN^{a}	Cov	PDB Hit	Associated GO Terms
1	0.44	0.4853	1.13	0.46	0.50	2zwmA	GO:0000156 GO:0000160 GO:0006355
2	0.43	0.4900	1.21	0.39	0.51	1xheB	GO:0000156 GO:0000160 GO:0006355
3	0.42	0.4786	1.18	0.39	0.49	3nhzC	GO:0006351 GO:0044117 GO:0006355 GO:0000160 GO:0003677 GO:0005886 GO:0006468 GO:0000156
4	0.38	0.4892	1.12	0.35	0.51	1zh4A	GO:0000156 GO:0000160 GO:0006355
5	0.38	0.8832	1.59	0.35	0.94	2gwrA	GO:0006468 GO:0006355 GO:0006351 GO:0005886 GO:0003677 GO:0000160 GO:0044117 GO:0000156 GO:0045893
6	0.34	0.7694	2.83	0.32	0.91	1ys6B	GO:0000287 GO:0006355 GO:0044110 GO:0005509 GO:0052572 GO:0005886 GO:0005829 GO:0003677 GO:0000156 GO:000 GO:0006351
7	0.33	0.5537	4.02	0.18	0.70	1s8nA	GO:0006355 GO:0005886 GO:0000156 GO:0000160 GO:0006351 GO:0031564 GO:0040007
8	0.30	0.5079	1.56	0.35	0.54	3crnA	GO:0000156 GO:0000160 GO:0006355
9	0.26	0.6117	4.06	0.22	0.77	2v0nA	GO:0005525 GO:0005737 GO:0000160 GO:0007049 GO:0007165 GO:0016740 GO:0030154 GO:0052621 GO:0000166 GO:004 GO:0000156 GO:0006355 GO:0009190 GO:0016849 GO:0035556
10	0.25	0.5507	4.40	0.16	0.77	1a2oA	GO:0006355 GO:0006935 GO:0005737 GO:0008984 GO:0000156 GO:0016787 GO:0000160

Consensus prediction of GO terms

**Molecular Function**	GO:0000156	GO:0003677		
**GO-Score**	0.93	0.64		
**Biological Process**	GO:0000160	GO:0044117	GO:0006468	GO:0045893
**GO-Score**	0.93	0.64	0.64	0.38
**Cellular Component**	GO:0005886			
**GO-Score**	0.64			

(a) Cscore^{GO} is a combined measure for evaluating global and local similarity between query and template protein. It's range is [0-1] and higher values indicate more confident predictions.
(b) TM-score is a measure of global structural similarity between query and template protein.
(c) RMSD^{a} is the RMSD between residues that are structurally aligned by TM-align.
(d) IDEN^{a} is the percentage sequence identity in the structurally aligned region.
(e) Cov represents the coverage of global structural alignment and is equal to the number of structurally aligned residues divided by length of the query protein.
(f) The second table shows a consensus GO terms amongst the top scoring templates. The GO-Score associated with each prediction is defined as the average weight of the GO term, where the weights are assigned [illegible] template.

[Click on S425338_results.tar.bz2 to download the tarball file including all modeling results listed on this page]

Please cite the following articles when you use the I-TASSER server:

1. Yang Zhang. I-TASSER: Fully automated protein structure prediction in CASP8. Proteins, 77 (Suppl 9): 100-113, 2009.
2. Ambrish Roy, Jianyi Yang, Yang Zhang. COFACTOR: an accurate comparative algorithm for structure-based protein function annotation. Nucleic Acids Research, 40: W471-W477, 2012.
3. Jianyi Yang, Yang Zhang. I-TASSER server: new development for protein structure and function predictions, Nucleic Acids Research, 43: W174-W181, 2015.
