# Supplementary material for: Prediction of Structure and Molecular Interaction with DNA of BvrR, a Virulence-Associated Regulatory Protein of Brucella
Source: Molecules. 2019 Aug 29;24(17):3137. doi: 10.3390/molecules24173137 (PMC6749498; doi:10.3390/molecules24173137)
Supplement: Supplementary file 1 [file molecules-24-03137-s001.zip › molecules-545148-revised-1-SM - final/Supplemental information S2.pdf]

Supplemental information S2

COACH results for BvrR

TM-SITE results for BvrR

Rank	C-score[a]	Cluster size[b]	Rep Templ[c]	Mult Templ[d]	Ligands[e]	Predicted binding site residues
1	0.45	12	3breA_BS01_MG	Download	MG(7),MN(3),BEF(2),..	14,15,16,58,60,107
2	0.19	2	4yn8A_BS01_MG	Download	MG(2)	14,16,58,109
3	0.18	2	1zdmA_BS02_XE	Download	XE(2)	13,14,23,56,57,58,84
4	0.16	1	3gwgA_BS01_MG	Download	MG(1)	19,107

a) **C-score:** is the confidence score of predicted binding site. C-score ranges [0-1], where a higher score indicates a more reliable prediction.

b) **Cluster size:** is the number of templates in a cluster.

c) **Rep Templ** presents a representative ligand-protein template from a cluster. The template names are: (PDBIDchain)_(BioLiP site #)_(Ligand ID).

d) **Mult Templ:** provides all ligand-protein complex structures in a cluster, where each ligand is separated by "TER".

e) **Ligands:** lists all ligands in a cluster. The numbers in the parentheses are the appearing times of the corresponding ligands.

S-SITE results for BvrR

Rank	C-score[a]	Cluster size[b]	Templates[c]	Ligands[d]	Predicted binding site residues
1	0.44	101	List	MG(39),BEF(29),MN(20),..	14,15,16,58,59,60,85,86,107
2	0.18	18	List	III(15),LA(1),SO4(1),..	88,89,90,91,93,94,97,101,102,103,104,105,106,113,117,120
3	0.12	2	List	NUC(2)	186,203,206,210,213,228,230,231,234
4	0.12	6	List	NUC(4),GLY(1),AZI(1)	134,167,168,169,189,190,191,192,194,195,199,200,201,203,204,207,208,211,230
5	0.12	1	List	BF2(1)	124

a) **C-score:** is the confidence score of predicted binding site. C-score ranges [0-1], where a higher score indicates a more reliable prediction.

b) **Cluster size:** is the number of templates in a cluster.

c) **Templates:** presents the list of templates in a cluster. The template names are: (PDBIDchain)_(BioLiP site #)_(Ligand ID).

d) **Ligands:** lists ligands in a cluster. The numbers in the parentheses are the appearing times for the corresponding ligands.

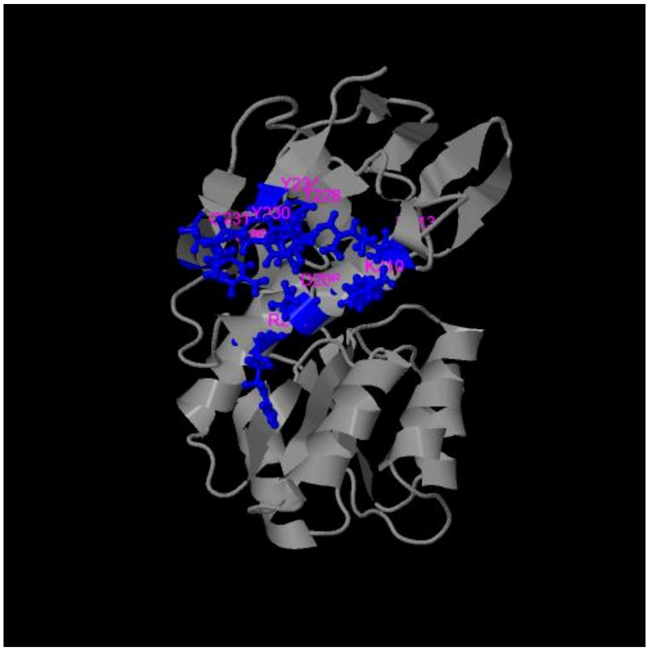

Figure S2.1 First Nucleic Acid binding site found with S-SITE.

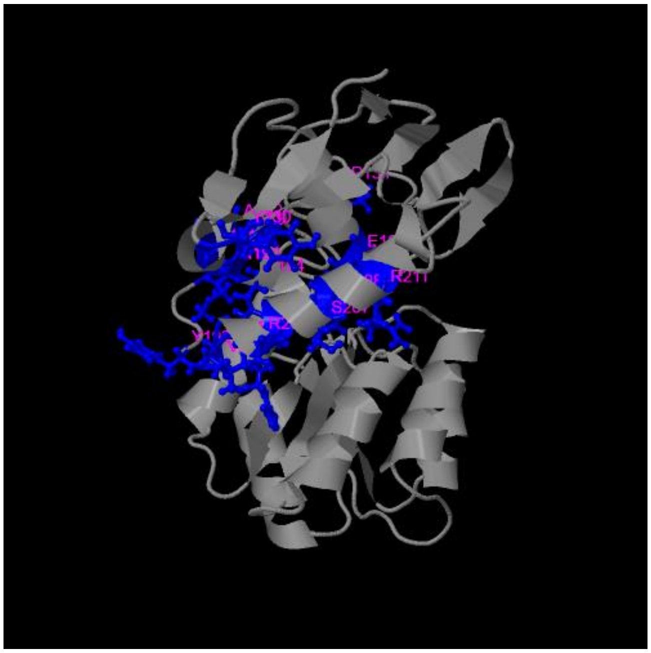

Figure S2.2 Second Nucleic Acid binding site found with S-SITE.

COFACTOR results for BvrR

Molecular Function (MF)

GO term	$Cscore^{GO}$	Name
GO:1901363	0.94	heterocyclic compound binding
GO:0097159	0.94	organic cyclic compound binding
GO:0003676	0.89	nucleic acid binding
GO:0003677	0.87	DNA binding
GO:0004871	0.79	signal transducer activity
GO:0000156	0.73	phosphorelay response regulator activity

Download full result of the above consensus prediction.

Click the graph to show a high resolution version.

(a) $Cscore^{GO}$ is the confidence score of predicted GO terms. $Cscore^{GO}$ values range in between [0-1]; where a higher value indicates a better confidence in predicting the function using the template.

(b) The graph shows the predicted terms within the Gene Ontology hierachy for Molecular Function. Confidently predicted terms are color coded by $Cscore^{GO}$:

[0.4,0.5) [0.5,0.6) [0.6,0.7) [0.7,0.8) [0.8,0.9) [0.9,1.0]

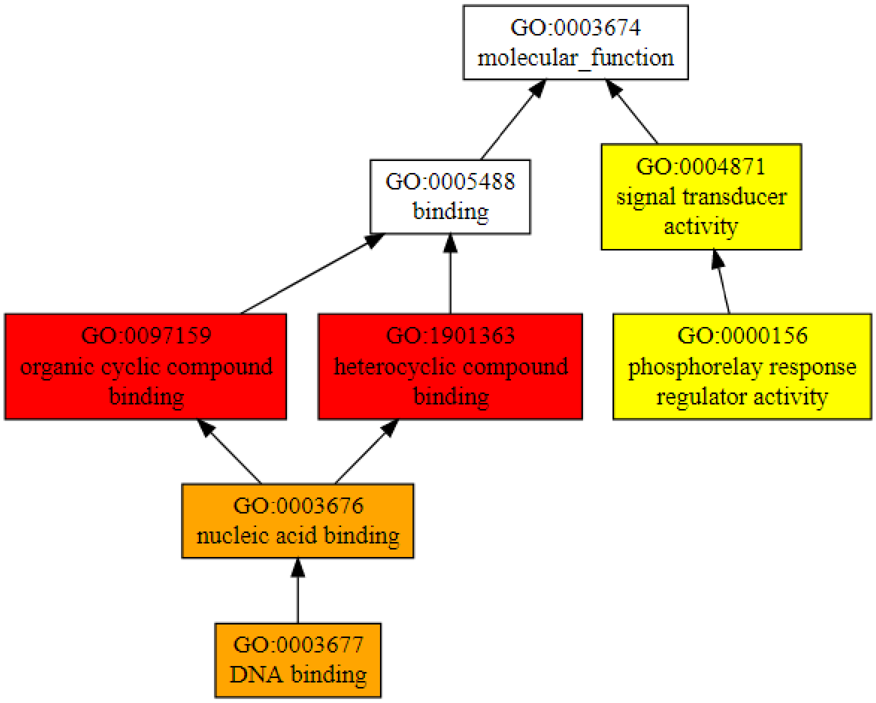

Biological Process (BP)

GO term	Cscore^{GO}	Name
GO:0065007	0.97	biological regulation
GO:0050789	0.97	regulation of biological process
GO:0050794	0.96	regulation of cellular process
GO:0009987	0.91	cellular process
GO:0007165	0.89	signal transduction
GO:0000160	0.88	phosphorelay signal transduction system
GO:0008152	0.71	metabolic process
GO:0044237	0.70	cellular metabolic process
GO:0071704	0.67	organic substance metabolic process
GO:0044238	0.61	primary metabolic process
GO:0019222	0.58	regulation of metabolic process
GO:0006807	0.58	nitrogen compound metabolic process
GO:0031323	0.56	regulation of cellular metabolic process
GO:0080090	0.55	regulation of primary metabolic process
GO:0060255	0.55	regulation of macromolecule metabolic process
GO:0009889	0.54	regulation of biosynthetic process
GO:0051171	0.53	regulation of nitrogen compound metabolic process
GO:0031326	0.53	regulation of cellular biosynthetic process
GO:0034641	0.52	cellular nitrogen compound metabolic process
GO:0010468	0.52	regulation of gene expression
GO:2000112	0.51	regulation of cellular macromolecule biosynthetic process
GO:0006355	0.50	regulation of transcription, DNA-templated

Download full result of the above consensus prediction.

Click the graph to show a high resolution version.

(a) Cscore^{GO} is the confidence score of predicted GO terms. Cscore^{GO} values range in between [0-1]; where a higher value indicates a better confidence in predicting the function using the template.

(b) The graph shows the predicted terms within the Gene Ontology hierachy for Biological Process. Confidently predicted terms are color coded by Cscore^{GO}:

[0.4,0.5) [0.5,0.6) [0.6,0.7) [0.7,0.8) [0.8,0.9) [0.9,1.0]

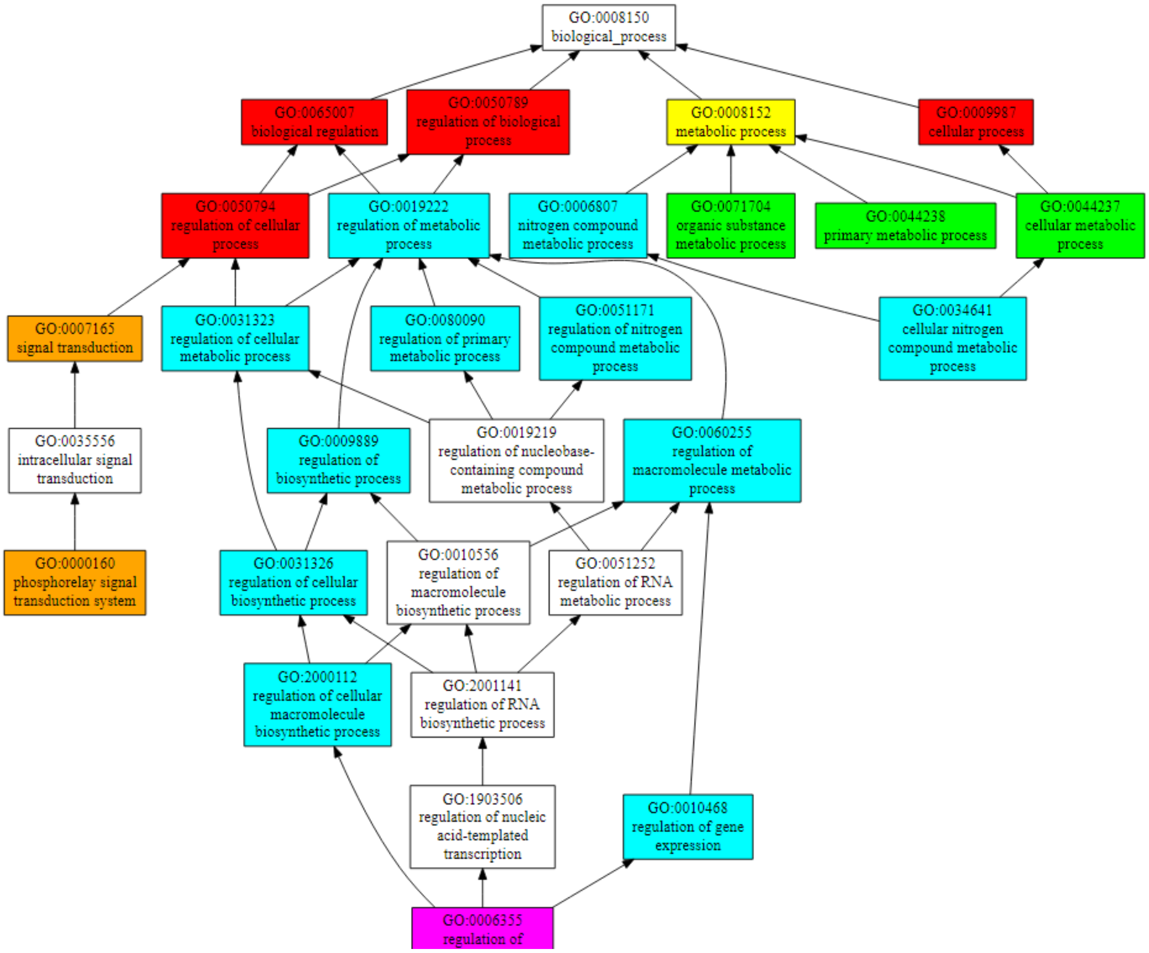

CATH results for BvrR

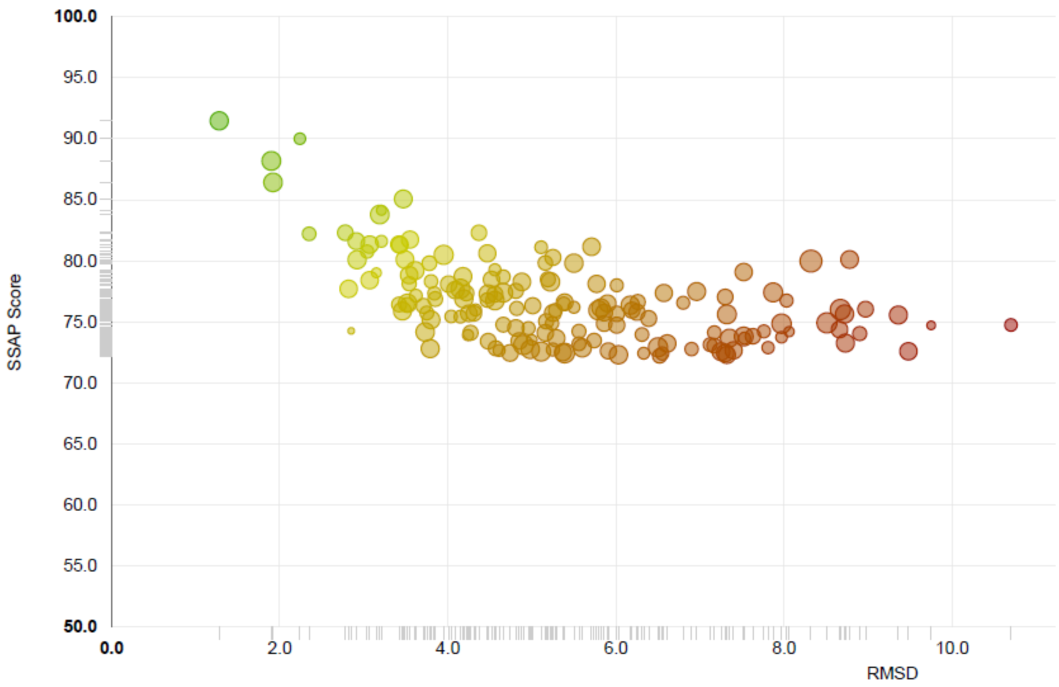

Domain	Length (AA residues)	Superfamily	RMSD (Å)	SSAP Score (0-100)	Hits	Actions
3w9sB00	117	3.40.50.2300	1.3	91.5	0 50 100 150 239	Show related domains
3c97A01	57	3.40.50.12330	2.2	90.0	0 50 100 150 239	Show related domains
3hzhA00	133	3.40.50.2300	1.9	88.2	0 50 100 150 239	Show related domains
1a2oA01	133	3.40.50.2300	1.9	86.4	0 50 100 150 239	Show related domains
3fkqA01	115	3.40.50.10850	3.5	85.1	0 50 100 150 239	Show related domains
4mloA02	46	1.10.10.1310	3.2	84.1	0 50 100 150 239	Show related domains
4mloA02	46	1.10.10.1310	3.2	84.1	0 50 100 150 239	Show related domains
