# Supplementary material for: Prediction of Structure and Molecular Interaction with DNA of BvrR, a Virulence-Associated Regulatory Protein of Brucella
Source: Molecules. 2019 Aug 29;24(17):3137. doi: 10.3390/molecules24173137 (PMC6749498; doi:10.3390/molecules24173137)
Supplement: Supplementary file 1 [file molecules-24-03137-s001.zip › molecules-545148-revised-1-SM - final/Supplemental Information S3.pdf]

PROCHECK

Ramachandran Plot

9257206

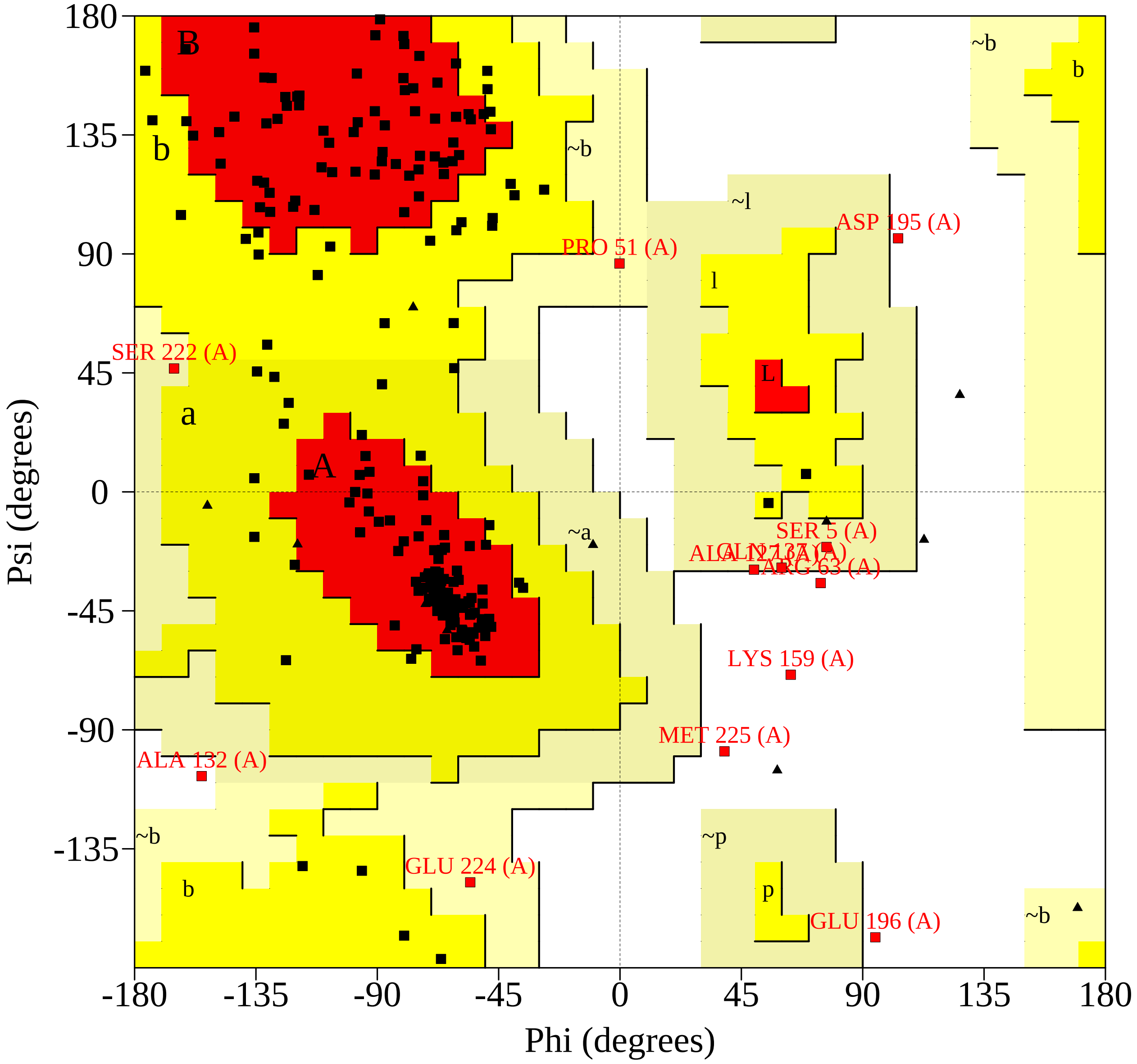

Plot statistics

Residues in most favoured regions [A,B,L]	163	75.5%
Residues in additional allowed regions [a,b,l,p]	42	19.4%
Residues in generously allowed regions [~a,~b,~l,~p]	5	2.3%
Residues in disallowed regions	6	2.8%
	----	------
Number of non-glycine and non-proline residues	216	100.0%
Number of end-residues (excl. Gly and Pro)	2	
Number of glycine residues (shown as triangles)	13	
Number of proline residues	8	

Total number of residues	239	

Based on an analysis of 118 structures of resolution of at least 2.0 Angstroms and R-factor no greater than 20%, a good quality model would be expected to have over 90% in the most favoured regions.

9257206_01.ps
