# Supplementary material for: Prediction of Structure and Molecular Interaction with DNA of BvrR, a Virulence-Associated Regulatory Protein of Brucella
Source: Molecules. 2019 Aug 29;24(17):3137. doi: 10.3390/molecules24173137 (PMC6749498; doi:10.3390/molecules24173137)

Supplemental information S4

Verify 3D results for BvrR

Verify 3D results

85.77% of the residues have averaged 3D-1D score >= 0.2

Pass

At least 80% of the amino acids have scored >= 0.2 in the 3D/1D profile.

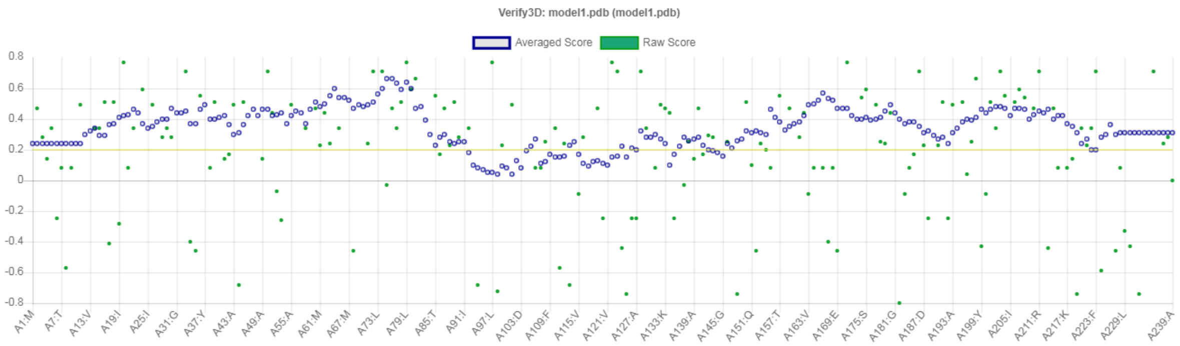

Supplement: Supplementary file 1 [file molecules-24-03137-s001.zip › molecules-545148-revised-1-SM - final/Supplemental information S4.pdf]
