# Supplementary material for: Prediction of Structure and Molecular Interaction with DNA of BvrR, a Virulence-Associated Regulatory Protein of Brucella
Source: Molecules. 2019 Aug 29;24(17):3137. doi: 10.3390/molecules24173137 (PMC6749498; doi:10.3390/molecules24173137)

Supplemental information S6

PROVE results for BvrR

Input file: **model1.pdb**

- **Job output**

Model 1: 49 buried outlier protein atoms, 5.6% **ERROR**

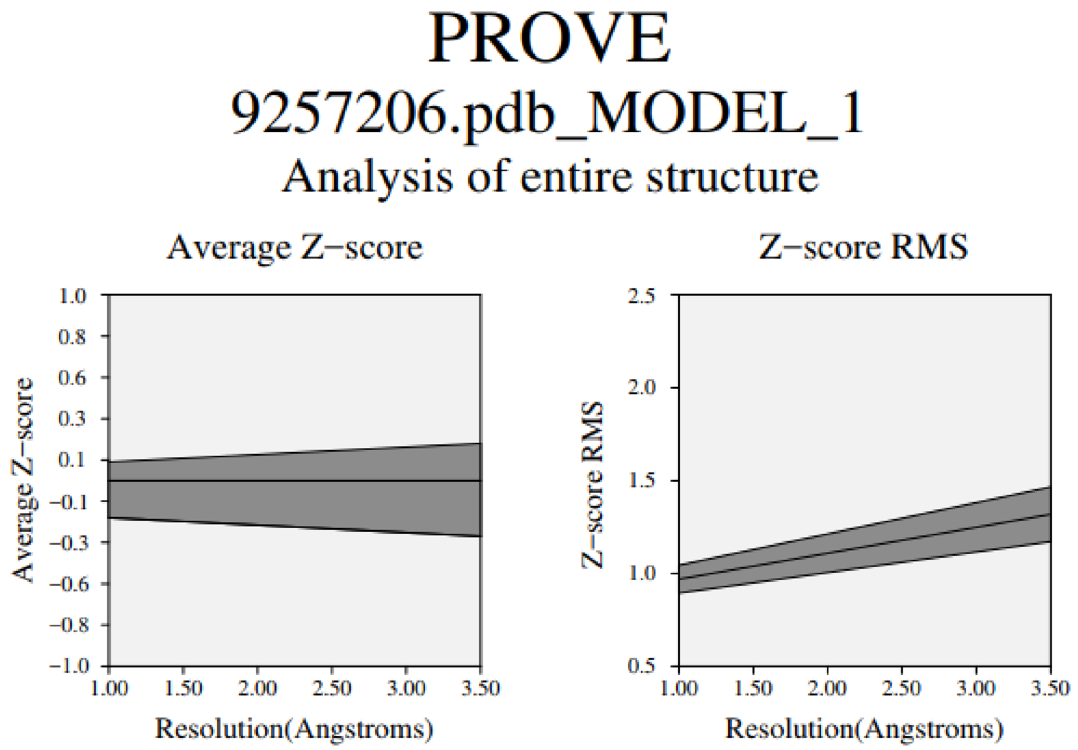

Supplement: Supplementary file 1 [file molecules-24-03137-s001.zip › molecules-545148-revised-1-SM - final/Supplemental information S6.pdf]
