# Supplementary material for: Prediction of Structure and Molecular Interaction with DNA of BvrR, a Virulence-Associated Regulatory Protein of Brucella
Source: Molecules. 2019 Aug 29;24(17):3137. doi: 10.3390/molecules24173137 (PMC6749498; doi:10.3390/molecules24173137)

Supplemental Information S7

ProSA results for BvrR

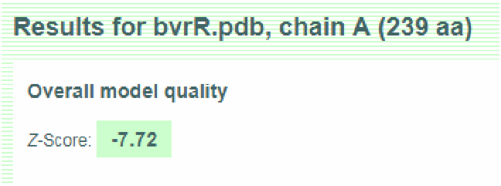
Results for bvrR.pdb, chain A (239 aa)

Overall model quality

Z-Score: -7.72

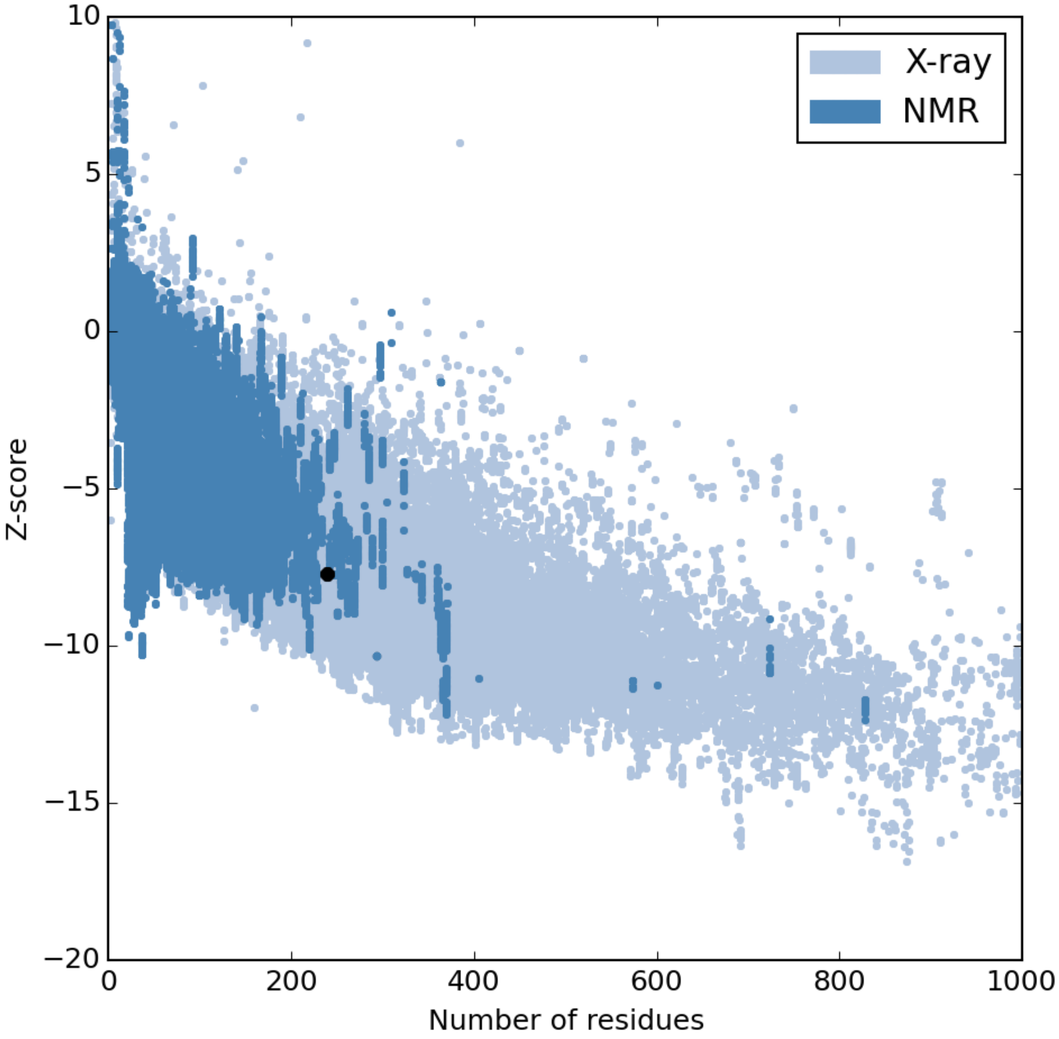

Local model quality

HELP

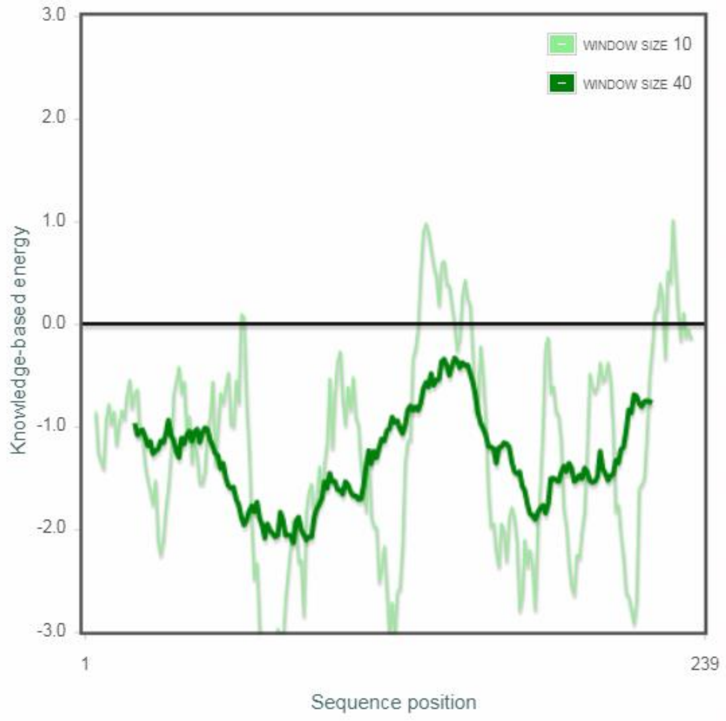

Supplement: Supplementary file 1 [file molecules-24-03137-s001.zip › molecules-545148-revised-1-SM - final/Supplemental Information S7.pdf]
