# Supplementary material for: Prediction of Structure and Molecular Interaction with DNA of BvrR, a Virulence-Associated Regulatory Protein of Brucella
Source: Molecules. 2019 Aug 29;24(17):3137. doi: 10.3390/molecules24173137 (PMC6749498; doi:10.3390/molecules24173137)
Supplement: Supplementary file 1 [file molecules-24-03137-s001.zip › molecules-545148-revised-1-SM - final/Supplemental information S8.pdf]

Supplemental information S8

Sequences of the 170 bp segments upstream the start codon of the 32 genes used for the Gibbs Sampling analysis. (The start codon is shown in bold letter).

Gene	Sequence
omp25a (BAB1_0722, BAB_RS19375)	CTCGACAGATTATCTCCACACAATGGGGCATTTCGTGCCGCAATTACCCTCGATATGTCACCCCTGTC AGCGCGGCATGGGCGGTTTACTCCCGATGCTGCCCGCCCGATAAGGGACCGCGCAAAACGTAATTT GTGTAAGGAGAATGCC**ATG**CGCACTCTTAAGTCTCTC
omp25d (BAB1_0115, BAB_RS16480)	TAAATTTCAAGTTAATCAAATATATATCAACTTTTTGTATAAATGAAAATCAAGACAATTCATTATTG TTGCTCATTTACTACATACATTCGACCAGATCATCACTATATGCTAGCCGCATTGCAACGCCATTCTT ATTAGGAGTTTTCA**ATG**ACGTTCAAAAATCTACTT
BAB1_0358, BAB_RS17640	CTTGGTCCACGTGTAACCCATAAGCCTCAACAGCCAGATACTCTGCTTATCGTGGATAAAAGTGGGT TGGCAGGGATTGTTCCGTGAAGGATATATTAACCGCGAATTTTCCGGTGTAAGAAAACTTTTTCGGTT CGGCGGACAGGGAAT**TTG**GATTTGCGGCGCATTGTT
BAB1_0589, BAB_RS18735	CTCACAAACGTCGAACCCAGCCGTGCATCAAGCAGCGGCCCATGCAAATTCAACTGTTCACCGCGC ACCGACCACCCCCGGTCTGGTCGCATTTCGTTAACCTTAAACGTTGTATCTCTATCCCTCTCCCATCA TCAATCTGGTTTTCAT**ATG**CAAACGACCCTTACCCGC
BAB1_2147, BAB_RS26155	GCGGTCGCGCCAAGGCGGGGATTTGACCTTTCGCCTTTTTTTGGCCTAAAAATTCCAAAACCTTGCAT GCGAAGAGAAATTGCCGGACGTTGATTTTGATCCTGTCCGCTTTGACTTGCACCCCAGCCGGATCGTT CGGATATTTCCGGG**ATG**GCTGGATCAGTTAAGGGT
htpX (BAB1_1821, BAB_RS24585)	CGTCGTTATTTTCGCTCATCGATAACATTCCAATTCTCGGCTTTGCCACAAATATTGAAGCGAAGCTT TTTCATACCCATCATATAGGCAGAAAGGCTTGCCGTGCAAAACCGTTTGACGGGCAATTGCCAGATT TGGAGACCGCCACGG**ATG**AATATGACGAAAACTGCC
moxR (BAB1_1573, BAB_RS23420)	CTTCCTTGCCGCCGGGGGTGCTTTTTATCATCCGACTCGGTTATTCCTGCGCAAATAGACTTAAAGCC ATGAAATAGTCACCCTTTCACGGCTTGTCAGTATTATATAGCGATTTATGAAGCAGTTCCGCCAGAG CATTGCGAGGTCCAT**ATG**AGCGTTGCCGTTACGCCT
clpB (BAB1_1868, BAB_RS24840)	TTTTTATTTGGATTTTCCGATGCCTGCCGGTCTTGCCGAAAGAGGGCAATATTCCCATATGCACTTTTG AGAAGAGCCCGCCCTTGTGGGGGGTCGTCACCCAACATCTCTCGGTGCCGCGGGACGGACCGAGAG GCAAACGGAGACATT**ATG**AATATCGAAAAATATACA
BAB2_1107, BAB_RS31625	GATCACCTATAAACCTCTGGGATGATGTTGAAATGCAGTTGCATCAACATGATCCCGGGCATTTTTTG CTTATGAATAACAGAATACTCGTACCTCCCTTTTGAAATGACCGCGAATTGATGTGGTAAATGACAG AGGCGGGGTTGAGAT**ATG**CCTAATTTGGCCAATCAG
s7 (BAB1_0505, BAB_RS18325)	TCCTGTGCGTGGTCATAGTAATAAAGAAGGTAGCAAGACTCAGCAAAATGACAATGGCTTTCCATC GCGGGCGGCCGCTTTGGCGCGAATTATCCGGTTAGGCTATCTGTTGGACTTGAAGCGACCATTTTTTT CATTTTCAGGGC**ATG**AAAATCTGTACCTCTATT
BAB1_1022, BAB_RS20820	TTCGTGGGGTTATCATCACAGGCAAGGGCTGATGGAGGCTGGCGCCCATCATATCCTGGAAAAGCCT TCCGATCTTCATGCTCTATTGCTGGAAATGGAAGAATTCTGAGCCTCGCCCTTTGAAATCCGTGACAC GGGACTGGATAATTC**ATG**CGTGACATTTTGAGCGAT

BAB1_1620, BAB_RS23635	AGGGACGCCCGTGCGCCAATTGTCCGCATAAGCCAAAAAATACACGATCTGGCCCAGGCGATGACA AAGCGCGCACACTTTATACTGCTGCCATTAATTGACTAGAATTCAAAGGCCTCCTAATGAATGACCA TTCCAAGGGGGGGCTTT**ATG**AAGTGTTATCTTATTAAT
fabG (BAB1_2043, BAB_RS25660)	GCTCGATCCTTATTGCCGCGCTTACGATCACCCCAATCTCTACGTGGTCGATGCGTCGTTCCTGCCAA CATCGGCAGCGGTCAATCCGGCGCTGACCATCGCCGCGCAAGCCTTGCGCGTCGCAGACCGTATCA AGCGGGAGGGTTTTGC**ATG**AGCCGTCAAAGACCTGTA
fabF (BAB1_0872, BAB_RS20090)	CGCTTGGCGCAATTTCCGTCTGGAAAGGCGAAGCCTTCGCCCCGCTGGATGCTTCGGAAAAGCCTGC CAGTGGCGCGGTCAGCGAAGCCATTGTCACCACCATCGGGGCAACCCGCGCTGAAGGCGCAGCAA AGCTGGTGAGAGCATAAG**ATG**ACGAAATATACAGACCAT
BAB1_0320, BAB_RS17460	TTAATTTACTCTTACGTCAATGTAACTCTTGCGAAGACAGTGAAACTCGACCACACTAAACAGCGAG GGGCTATCCGGGGGCGGATGGCTGGGAGATGGGCGGGCGCCCTAGCGGCCTGCACAAAGGACGGA GCGATCCGGGGAGAAGAC**ATG**GCAAAAAAGGTGACTGGG
cfa (BAB1_0476, BAB_RS18195)	TGTTGCTTTGGCATTTCTTCCAGGGCCGGTTGATTGACGGAAAACTGCCTGGAAATTTGACGGCAGA GGGGCTTCTTTTGATAATGCCGCCTGCCGGGCAGCAGCATGGATGTATGCACCTGCTTCGTTCCCAA AGAGGAGATGGGGCCG**ATG**TATGGAATGCTGCGTACA
BAB1_1357, BAB_RS22425	GTTTACCGTTCTCAATCGCGGTGTGACGCTTGGCACGCGCACCAAGGACGATGTGGATATGGATGAG TTGCAAAGCCTGATGGCAGGCGGGCAGGAGCTTGCCGATCTTACCGCTGAACTGGGCGGGCGAGTA TAACCGGAGAACATGGA**ATG**GGCGGACAGTTGAAGGGT
nirK (BAB2_0943, BAB_RS30770)	AATATAATTTAACTATCTGATTTTATTATGGAAATATTTCCATATCGAGCGCCTTATATTGATTGCGGT CAAGGAAGCGCCATGCGGCATCGCCTAGAAAGGAGGGGACAGAGCCAATGGCTCTTGGTCCTATCG CAAAGGAGAGAAACG**ATG**GCCGACCAGATACAAGTC
norC (BAB2_0955, BAB_RS30825)	CTGCATCTTTGCCTATTCGCAAAGAAGGGTTCTGCGCGACCGATAGAATGGAAGCATCCCGCGGGG CCGGCGAGGGGAATTGCCCGGCCGCATTTTCTGCGGGGGATTGGCTGCCGGAGCGATGGTTCCGGCC AATGAAAGGACGAAGGG**ATG**GCAGAACGCCTAACCAAG
nosZ (BAB2_0928, BAB_RS30695)	CGAAAAATTTGCAGCCCGCAATGCACCTCTCGCAGCGGCTGCCGAACGTGGCGGCGGTCCAGCCAG GCCTATCATCTCGGTCAAGGGAAAGCCGGTGGAAACACCCGTCAATCCCGGTCCCGCGGCCTGATTT TTTCAAAGGAGAAAAGT**ATG**ACTGAAGAAACCAGGAAA
BAB2_0863, BAB_RS30420	TACCTACGCAATTCGCAAGCCGCACTGGAAAACGCCGGAAGGGTCGGCCGATTTTGAGCCGTTCAA CTGGGAAAAGGAAGGCCGGATGCCGGGTTCCCAGCAAGCCGCAGGCTGAAAAGCCCGTCCTGCAA AAGCTATGGAGACATCTTA**ATG**TCAAGCTCATCAGATGCG
BAB1_0383, BAB_RS17760	GGCCCATGCGCGTGAAATCTATTTGCAGGCTGGCCGCTCCCATGCCATGCCGCCCGCCAACGTTACC GGCGTGACGGACGAGGAACGCCAGCTTCTGGCCTCCTGGTATCAATCCGTGACGACAGGCCCAAAA GAGGGGACCAAGACCGA**ATG**ACAAAGCTTCTCATCCGC
hPr-K (BAB1_2094, BAB_RS25910)	CGATCAGCCGGCAGATCATCGAGGCGCATGGCGGCGCGCTGACGGCGGAAAATATTACCGATCCGG ATAAGCCGGATATTTTCAAGGGTGCCCGCTTTATCGTTGATCTTCCGGCCAGCGCATGACCGTGAAA AGTGGATATCTGAACCT**GTG**ACGCCCGAGGAGGAAAGA
BAB1_2091,	CGGGTTAAACGATTTAAAAAAATTTCAAAAATTTTAAATCGATTAAATATTTGAATTTTCTCAATTAA

BAB_RS25895	TCTGTTTCTGACTGCCATCCCCCATGAAAGCCAAACCAGGGGGACGCATTTTTTAGTTAGTCAAACA<br>GACGGCGGAGACATC**ATG**AAAGAGACCGGCATCCAC
vjbR<br>(BAB2_0118,<br>BAB_RS26915)	AATTAAGTAATTGCCGGTTGCCAACGACTATATTTTTTTTAAGTCACTAATAAGCGATTGAAGGCCTC<br>TGAGGGGAGGCGATTCTCTCGCAAGCAGTTATAGAGATACCAGAGCGAAGCCCGCATGGTTTCATC<br>ACCAAGGATATTTCCA**ATG**AGTCTTGATCTCGTTCAT
exoR<br>(BAB1_0891,<br>BAB_RS20180)	GAGCAGAAAAAATTCTGCCCGTTTGCCTGTCGCCTCCGCCGAAGCGAGCTGAGCTTTTTTCCGTCAA<br>GATCGGGTCTCACTGTTGACGAAAAGCAGGCGCAACCTGCCTTTCATGATCTTTTCCGTTCCATATGA<br>AAGACCGGCGTTGTG**ATG**CGTAGTCGCAGTTTTTCA
ompR<br>(BAB2_0762,<br>BAB_RS29960)	AACGCCTATGCGGGGAAATCAGCCAGTAGGCTGATTTCTGATCCGGCTTCGATGGAAAATGCGCCA<br>AAACAAATAGTTAGAGCAGTTCCGCCGTTTCTGTTAAAATAGGAAACGCTACAATACCCACCCCAA<br>CGGCAATGGAACAGCAAA**TTG**AAAGAAGACGCCCACATT
motB<br>(BAB2_1103,<br>BAB_RS31605)	TCTGGAAGCATTGCAGGCCCATGGGGTGCTTTTGCCCTCCGGTTGCCGCTTTTCAGAGACGGCATCG<br>AACGAACTTTCCTTTTCCTTTGGCGAACGGATGCTCGAAACGCGCATCGCGCCGCTTCTTGAAGAAC<br>TGAAGGCTGCGGTGAA**ATG**AACATTGAGCCCGAAACG
malK<br>(BAB1_0241,<br>BAB_RS17050)	TCGATTATACGGCCGAGTTCACCATCGATTGGCGCGGCATGTGCGCGCTTGCCGTGGTGATGATTGTT<br>CCGGCGCTGGCGCTCACCTTCGTCATCCAGAAACACCTCGTTTCCGGCCTGACTTTCGGCGCGGTTA<br>AAGGTTGAAATTGTA**ATG**GCACAGCTTTCCATCAAA
BAB1_1601,<br>BAB_RS23550	TTCGTTGCTTAACAGAAGGACGCTGTCGTTCGCTTCAAGGCAAATGACGACTATTGGGGCGGACGCC<br>CGAAGATCGACGATCTGGTCTTCGCGATCACGACCGATCCTGCTGTTCGCGCGCAGAAGCTCAAGGC<br>CGGCGAATGCCAGCTT**ATG**TCCTATCCGGCTCCGGCG
BAB1_1397,<br>BAB_RS22600	AATATCTTTATTCCATAGAAACATGGGAAAAGCTGTTCTCGTCCGCTGAAAATTGCGCTAAACCACC<br>ATCGTCAATAGCTGGGTTTGAGGAGACTTCGCTATTACAAGTTCCGCTGCAAGAGGCGGATAGTCTA<br>ATTGAGGAGACTTCCC**GTG**TTGGACTGGGAAAATGTA
BAB1_0666,<br>BAB_RS19125	TCTGCCGCATTATCTCTTTTTTGCAGATACACCCCGCTTGCTCCATTCGCGTGGCAAGACTATGGTGC<br>AACGCCTCACGCATAATCCGATGAAGCCCGAAGGGGGCTTTGCTGCGTGGGGTCCCGTCTATCTGTT<br>TCAGGAGTTCACGA**ATG**CTGAAGGGCTCAATTACCG
