# Supplementary material for: Prediction of Structure and Molecular Interaction with DNA of BvrR, a Virulence-Associated Regulatory Protein of Brucella
Source: Molecules. 2019 Aug 29;24(17):3137. doi: 10.3390/molecules24173137 (PMC6749498; doi:10.3390/molecules24173137)
Supplement: Supplementary file 1 [file molecules-24-03137-s001.zip › molecules-545148-revised-1-SM - final/Supplemental Information S9/Supplemental Information S9.pdf]

Supplementary Information S9.

Table S1 | Motif obtained with *Gibbs Recursive Sampling* with the most optimal result.

Left Localization	Motif	Right Localization	Probability	Forward motif (F) o Reverse motif (R)	Gene	Gene ID
75	cgaag AGAAATTGCC ggacg	84	0.96	F	Lipoprotein	BAB1_2147
162	atgac GAAAACTGCC -------	171	0.93	F	Heat Shock Protein	BAB1_1821
67	taagc AAAAAATGCC cggga	58	0.97	R	Cell wall surface protein	BAB2_1107
17	cagac GGAAATTGCG ccaag	8	0.96	R	Acyltransferase FabF	BAB1_0872
19	cctgg AAGAAATGCC aaagc	10	0.90	R	Methyltransferase Cfa	BAB1_0476
40	tgacg GAAAACTGCC tggaa	49	0.93	F	Methyltransferase Cfa	BAB1_0476
75	gcgag GGGAATTGCC cggcc	84	0.86	F	Nitric oxide reductase NorC	BAB2_0955
98	cccgc AGAAAATGCG gccgg	89	0.97	R	Nitric oxide reductase NorC	BAB2_0955
122	taact AAAAAATGCG tcccc	113	0.97	R	Phosphoenolpyruvate carboxykinase	BAB1_2091
169	----tg AAAAACTGCG actac	160	0.94	R	Negative exopolysaccharide regulator ExoR	BAB1_0891
54	tcgat GGAAAATGCG ccaaa	63	0.96	F	Transcriptional regulator OmpR	BAB2_0762
48	ccgct GAAAATTGCG ctaaa	57	0.96	F	Transcriptional regulator GntR	BAB1_1397

Table S2 | Motif obtained with Gibbs Recursive Sampling with the result with a probability greater than 50%.

Left Localization	Motif	Right Localization	Probability	Forward motif (F) o Reverse motif (R)	Gene	Gene ID
36	gcggc ACGAAATGCC ccatt	27	0.52	R	Outer membrane protein Omp25a	BAB1_0722
97	ggtta ACGAAATGCG accag	88	0.52	R	L, D transpeptidase	BAB1_0589
75	cgaag AGAAATTGCC ggacg	84	0.96	F	Cell wall hydrolase	BAB1_2147
162	atgac GAAAACTGCC -------	171	0.79	F	Heat shock protein HtpX	BAB1_1821
67	taagc AAAAAATGCC cggga	58	0.96	R	Cell wall surface protein	BAB2_1107
17	cagac GGAAATTGCG ccaag	8	0.97	R	Acyltransferase FabF	BAB1_0872
19	cctgg AAGAAATGCC aaagc	10	0.87	R	Methyltransferase Cfa	BAB1_0476
40	tgacg GAAAACTGCC tggaa	49	0.81	F	Methyltransferase Cfa	BAB1_0476
75	gcgag GGGAATTGCC cggcc	84	0.87	F	Nitric oxide reductase NorC	BAB2_0955
98	cccgc AGAAAATGCG gccgg	89	0.99	R	Nitric oxide reductase NorC	BAB2_0955
16	ggctt GCGAATTGCG taggt	7	0.53	R	Glutaminase R	BAB2_0863
122	taact AAAAAATGCG tcccc	113	0.97	R	Phosphoenolpyruvate carboxykinase	BAB1_2091
169	tg AAAAACTGCG actac	160	0.82	R	Negative exopolysaccharide regulator ExoR	BAB1_0891
54	tcgat GGAAAATGCG ccaaa	63	0.96	F	Transcriptional regulator OmpR	BAB2_0762
48	ccgct GAAAATTGCG ctaaa	57	0.95	F	Transcriptional regulator GntR	BAB1_1397

Table S3 | Motif obtained with *Gibbs Recursive Sampling* limiting to three motifs per sequence and to three different motifs in total.

Left Localization	Motif	Right Localization	Probability	Forward motif (F) o Reverse motif (R)	Gene	Gene ID
37	cggtc GCGCAGAACC cttct	28	0.90	R	Nitric oxide reductase NorC	BAB2_0955
102	atccc CCGCAGAAAA tgcgg	93	0.87	R	Nitric oxide reductase NorC	BAB2_0955
133	tcatt GGCCGGAACC atcgc	124	0.94	R	Nitric oxide reductase NorC	BAB2_0955
9	tcagc CGGCAGATCA tcgag	18	0.59	F	Serine kinase of the HPr protein	BAB1_2094
43	gctga CGGCGGAAAA tatta	52	0.91	F	Serine kinase of the HPr protein	BAB1_2094
116	gcgct GGCCGGAAGA tcaac	107	0.78	R	Serine kinase of the HPr protein	BAB1_2094
74	gccag CGCCGGAACA atcat	65	0.94	R	ABC transporter	BAB1_0241
117	agtca GGCCGGAAAC gaggt	108	0.91	R	ABC transporter	BAB1_0241
