# Supplementary material for: Prediction of Structure and Molecular Interaction with DNA of BvrR, a Virulence-Associated Regulatory Protein of Brucella
Source: Molecules. 2019 Aug 29;24(17):3137. doi: 10.3390/molecules24173137 (PMC6749498; doi:10.3390/molecules24173137)
Supplement: Supplementary file 1 [file molecules-24-03137-s001.zip › molecules-545148-revised-1-SM - final/Supplemental Information S9/Table S4.pdf]

Table S4. Sequences of genes with the AAHTGC motif. (The start codon is shown remarked in yellow and the motif remarked in purple)

Gene ID	Sequence
BAB1_2147	GCGGTCGCGCCAAGGCGGGGATTTGACCTTTCGCCTTTTTTTGGCCTAAAAATTCCAAAACCTTGCATGCGAAGAGAAATTGCCGGACGTTGATTTTGATCCTGTCCGCTTTGACTTGCACCCCAGCCGGATCGTTCGGATATTTCCGGGATGGCTGGATCAGTTAAGGGT<br>CGCCAGCGCGGTTCCGCCCCTAAACTGGAAAGCGGAAAAAAACCGGATTTTTAAGGTTTTGGAACGTACGCTTCTCTTTAACGGCCTGCAACTAAAACTAGGACAGGCGAAACTGAACGTGGGGTCGGCCTAGCAAGCCTATAAAGGCCCTACCGACCTAGTCAATTCCCA
BAB1_1821	CGTCGTTATTTTCGCTCATCGATAACATTCCAATTCTCGGCTTTGCCACAAATATTGAAGCGAAGCTTTTTCATACCCATCATATAGGCAGAAAGGCTTGCCGTGCAAAACCGTTTGACGGGCAATTGCCAGATTTGGAGACCGCCACGGATGAATATGACGAAAACTGCC<br>GCAGCAATAAAAGCGAGTAGCTATTGTAAGGTTAAGAGCCGAAACGGTGTTTATAACTTCGCTTCGAAAAAGTATGGGTAGTATATCCGTCTTTCCGAACGGCACGTTTTGGCAAACTGCCCGTTAACGGTCTAAACCTCTGGCGGTGCCTACTTATACTGCTTTTGACGG
BAB2_1107	GATCACCTATAAACCTCTGGGATGATGTTGAAATGCAGTTGCATCAACATGATCCCGGGCATTTTTTGCTTATGAATAACAGAATACTCGTACCTCCCTTTTGAAATGACCGCGAATTGATGTGGTAAATGACAGAGGCGGGGTTGAGATATGCCTAATTTGGCCAATCAG<br>CTAGTGGATATTTGGAGACCCTACTACAACTTTACGTCAACGTAGTTGTACTAGGGCCCGTAAAAAACGAATACTTATTGTCTTATGAGCATGGAGGGAAAACTTTACTGGCGCTTAACTACACCATTTACTGTCTCCGCCCCAACTCTATACGGATTAAACCGGTTAGTC
BAB1_0872	CGCTTGGCGCAATTTCCGTCTGGAAAGGCGAAGCCTTCGCCCCGCTGGATGCTTCGGAAAAGCCTGCCAGTGGCGCGGTCAGCGAAGCCATTGTCACCACCATCGGGGCAACCCGCGCTGAAGGCGCAGCAAAGCTGGTGAGAGCATAAGATGACGAAATATACAGACCAT<br>GCGAACCGCGTTAAAGGCAGACCTTTCCGCTTCGGAAGCGGGGCGACCTACGAAGCCTTTTCGGACGGTCACCGCGCCAGTCGCTTCGGTAACAGTGGTGGTAGCCCCGTTGGGCGCGACTTCCGCGTCGTTTCGACCACTCTCGTATTCTACTGCTTTATATGTCTGGTA
BAB1_0476	TGTTGCTTTGGCATTTCTTCCAGGGCCGGTTGATTGACGGAAAACTGCCTGGAAATTTGACGGCAGAGGGGCTTCTTTTGATAATGCCGCCTGCCGGGCAGCAGCATGGATGTATGCACCTGCTTCGTTCCCAAAGAGGAGATGGGGCCGATGTATGGAATGCTGCGTACA<br>ACAACGAAACCGTAAAGAAGGTCCCGGCCAACTAACTGCCTTTTGACGGACCTTTAAACTGCCGTCTCCCCGAAGAAAACTATTACGGCGGACGGCCCGTCGTCGTACCTACATACGTGGACGAAGCAAGGGTTTCTCCTCTACCCCGGCTACATACCTTACGACGCATGT
BAB2_0955	CTGCATCTTTGCCTATTCGCAAAGAAGGGTTCTGCGCGACCGATAGAATGGAAGCATCCCGCGGGGCCGGCGAGGGGAATTGCCCGGCCGCATTTTCTGCGGGGGATTGGCTGCCGGAGCGATGGTTCCGGCCAATGAAAGGACGAAGGGATGGCAGAACGCCTAACCAAG<br>GACGTAGAAACGGATAAGCGTTTCTTCCCAAGACGCGCTGGCTATCTTACCTTCGTAGGGCGCCCCGGCCGCTCCCCTTAACGGGCCGGCGTAAAAGACGCCCCCTAACCGACGGCCTCGCTACCAAGGCCGGTTACTTTCCTGCTTCCCTACCGTCTTGCGGATTGGTTC
BAB1_2091	CGGGTTAAACGATTTAAAAAAATTTCAAAAATTTTAAATCGATTAAATATTTGAATTTTCTCAATTAATCTGTTTCTGACTGCCATCCCCCATGAAAGCCAAACCAGGGGGACGCATTTTTTAGTTAGTCAAACAGACGGCGGAGACATCATGAAAGAGACCGGCATCCAC<br>GCCCAATTTGCTAAATTTTTTTAAAGTTTTTAAAATTTAGCTAATTTATAAACTTAAAAGAGTTAATTAGACAAAGACTGACGGTAGGGGGTACTTTCGGTTTGGTCCCCCTGCGTAAAAAATCAATCAGTTTGTCTGCCGCCTCTGTAGTACTTTCTCTGGCCGTAGGTG
BAB1_0891	GAGCAGAAAAAATTCTGCCCGTTTGCCTGTCGCCTCCGCCGAAGCGAGCTGAGCTTTTTTCCGTCAAGATCGGGTCTCACTGTTGACGAAAAGCAGGCGCAACCTGCCTTTCATGATCTTTTCCGTTCCATATGAAAGACCGGCGTTGTGATGCGTAGTCGCAGTTTTTCA<br>CTCGTCTTTTTTAAGACGGGCAAACGGACAGCGGAGGCGGCTTCGCTCGACTCGAAAAAAGGCAGTTCTAGCCCAGAGTGACAACTGCTTTTCGTCCGCGTTGGACGGAAAGTACTAGAAAAGGCAAGGTATACTTTCTGGCCGCAACACTACGCATCAGCGTCAAAAAGT
BAB2_0762	AACGCCTATGCGGGGAAATCAGCCAGTAGGCTGATTTCTGATCCGGCTTCGATGGAAAATGCGCCAAAACAAATAGTTAGAGCAGTTCCGCCGTTTCTGTTAAAATAGGAAACGCTACAATACCCACCCCAACGGCAATGGAACAGCAAATTGAAAGAAGACGCCCACATT<br>TTGCGGATACGCCCCTTTAGTCGGTCATCCGACTAAAGACTAGGCCGAAGCTACCTTTTACGCGGTTTTGTTTATCAATCTCGTCAAGGCGGCAAAGACAATTTTATCCTTTGCGATGTTATGGGTGGGGTTGCCGTTACCTTGTCGTTTAACTTTCTTCTGCGGGTGTAA
BAB1_1397	AATATCTTTATTCCATAGAAACATGGGAAAAGCTGTTCTCGTCCGCTGAAAATTGCGCTAAACCACCATCGTCAATAGCTGGGTTTGAGGAGACTTCGCTATTACAAGTTCCGCTGCAAGAGGCGGATAGTCTAATTGAGGAGACTTCCCGTGTTGGACTGGGAAAATGTA<br>TTATAGAAATAAGGTATCTTTGTACCCTTTTCGACAAGAGCAGGCGACTTTTAACGCGATTTGGTGGTAGCAGTTATCGACCCAAACTCCTCTGAAGCGATAATGTTCAAGGCGACGTTCTCCGCCTATCAGATTAACTCCTCTGAAGGGCACAACCTGACCCTTTTACAT
BAB1_0722	CTCGACAGATTATCTCCACACAATGGGGCATTTCGTGCCGCAATTACCCTCGATATGTCACCCCTGTCAGCGCGGCATGGGCGGTTTACTCCCGATGCTGCCCGCCCGATAAGGGACCGCGCAAAACGTAATTTGTGTAAGGAGAATGCCATGCGCACTCTTAAGTCTCTC<br>GAGCTGTCTAATAGAGGTGTGTTACCCCGTAAAGCACGGCGTTAATGGGAGCTATACAGTGGGGACAGTCGCGCCGTACCCGCCAAATGAGGGCTACGACGGGCGGGCTATTCCCTGGCGCGTTTTGCATTAAACACATTCCTCTTACGGTACGCGTGAGAATTCAGAGAG
BAB1_0589	CTCACAAACGTCGAACCCAGCCGTGCATCAAGCAGCGGCCCATGCAAATTCAACTGTTCACCGCGCACCGACCACCCCCGGTCTGGTCGCATTTCGTTAACCTTAAACGTTGTATCTCTATCCCTCTCCCATCATCAATCTGGTTTTCATATGCAAACGACCCTTACCCGC<br>GAGTGTTTGCAGCTTGGGTCGGCACGTAGTTCGTCGCCGGGTACGTTTAAGTTGACAAGTGGCGCGTGGCTGGTGGGGGCCAGACCAGCGTAAAGCAATTGGAATTTGCAACATAGAGATAGGGAGAGGGTAGTAGTTAGACCAAAAGTATACGTTTGCTGGGAATGGGCG
BAB2_0863	TACCTACGCAATTCGCAAGCCGCACTGGAAAACGCCGGAAGGGTCGGCCGATTTTGAGCCGTTCAACTGGGAAAAGGAAGGCCGGATGCCGGGTTCCCAGCAAGCCGCAGGCTGAAAAGCCCGTCCTGCAAAAGCTATGGAGACATCTTAATGTCAAGCTCATCAGATGCG<br>ATGGATGCGTTAAGCGTTCGGCGTGACCTTTTGCGGCCTTCCCAGCCGGCTAAAACTCGGCAAGTTGACCCTTTTCCTTCCGGCCTACGGCCCAAGGGTCGTTCGGCGTCCGACTTTTCGGGCAGGACGTTTTCGATACCTCTGTAGAATTACAGTTCGAGTAGTCTACGC
